# Evaluation of two short standardised regimens for the treatment of rifampicin-resistant tuberculosis (STREAM stage 2): an open-label, multicentre, randomised, non-inferiority trial

**DOI:** 10.1016/S0140-6736(22)02078-5

**Published:** 2022-11-08

**Authors:** Ruth L Goodall, Sarah K Meredith, Andrew J Nunn, Adamu Bayissa, Anuj K Bhatnagar, Gay Bronson, Chen-Yuan Chiang, Francesca Conradie, Meera Gurumurthy, Bruce Kirenga, Nana Kiria, Daniel Meressa, Ronelle Moodliar, Gopalan Narendran, Nosipho Ngubane, Mohammed Rassool, Karen Sanders, Rajesh Solanki, S Bertel Squire, Gabriela Torrea, Bazarragchaa Tsogt, Elena Tudor, Armand Van Deun, ID Rusen, for the STREAM study collaborators

**Affiliations:** Medical Research Council Clinical Trials Unit at UCL, University College London, London, UK; Medical Research Council Clinical Trials Unit at UCL, University College London, London, UK; St Peter’s Tuberculosis Specialized Hospital and Global Health Committee, Addis Ababa, Ethiopia; Medical Research Council Clinical Trials Unit at UCL, University College London, London, UK; Armauer Hansen Research Institute, Addis Ababa, Ethiopia; Rajan Babu Institute for Pulmonary Medicine & Tuberculosis, Delhi, India; Vital Strategies, New York, NY, USA; Department of Internal Medicine, Wan Fang Hospital, Taipei Medical University, Taipei, Taiwan; International Union against Tuberculosis and Lung Disease, Paris, France; Empilweni TB Hospital, Eastern Cape, South Africa; Vital Strategies, Singapore; Makerere University Lung Institute, Mulago Hospital, Kampala, Uganda; National Center for Tuberculosis and Lung Diseases, Tbilisi, Georgia; Tuberculosis Specialized Hospital and Global Health Committee, Addis Ababa, Ethiopia; Tuberculosis & HIV Investigative, Doris Goodwin Hospital, Pietermaritzburg, South Africa; National Institute for Research in Tuberculosis, Chennai, India; King Dinuzulu Hospital Complex, Durban, South Africa; Clinical HIV Research Unit, Helen Joseph Hospital, Department of Internal Medicine, University of the Witwatersrand, Johannesburg, South Africa; Medical Research Council Clinical Trials Unit at UCL, University College London, London, UK; B J Medical College, Ahmedabad, India; Department of Clinical Sciences, Liverpool School of Tropical Medicine, Liverpool, UK; Institute of Tropical Medicine, Antwerp, Belgium; Mongolian Tuberculosis Coalition, Ulaanbaatar, Mongolia; Institute of Phthisiopneumology Chiril Draganiuc, Chisinau, Moldova; Institute of Tropical Medicine, Antwerp, Belgium; Vital Strategies, New York, NY, USA

## Abstract

**Background:**

The STREAM stage 1 trial showed that a 9-month regimen for the treatment of rifampicin-resistant tuberculosis was non-inferior to the 20-month 2011 WHO-recommended regimen. In STREAM stage 2, we aimed to compare two bedaquiline-containing regimens with the 9-month STREAM stage 1 regimen.

**Methods:**

We did a randomised, phase 3, non-inferiority trial in 13 hospital clinics in seven countries, in individuals aged 15 years or older with rifampicin-resistant tuberculosis without fluoroquinolone or aminoglycoside resistance. Participants were randomly assigned 1:2:2:2 to the 2011 WHO regimen (terminated early), a 9-month control regimen, a 9-month oral regimen with bedaquiline (primary comparison), or a 6-month regimen with bedaquiline and 8 weeks of second-line injectable. Randomisations were stratified by site, HIV status, and CD4 count. Participants and clinicians were aware of treatment-group assignments, but laboratory staff were masked. The primary outcome was favourable status (negative cultures for *Mycobacterium tuberculosis* without a preceding unfavourable outcome) at 76 weeks; any death, bacteriological failure or recurrence, and major treatment change were considered unfavourable outcomes. All comparisons used groups of participants randomly assigned concurrently. For non-inferiority to be shown, the upper boundary of the 95% CI should be less than 10% in both modified intention-to-treat (mITT) and per-protocol analyses, with prespecified tests for superiority done if non-inferiority was shown. This trial is registered with ISRCTN, ISRCTN18148631.

**Findings:**

Between March 28, 2016, and Jan 28, 2020, 1436 participants were screened and 588 were randomly assigned. Of 517 participants in the mITT population, 133 (71%) of 187 on the control regimen and 162 (83%) of 196 on the oral regimen had a favourable outcome: a difference of 11·0% (95% CI 2·9–19·0), adjusted for HIV status and randomisation protocol (p<0·0001 for non-inferiority). By 76 weeks, 108 (53%) of 202 participants on the control regimen and 106 (50%) of 211 allocated to the oral regimen had an adverse event of grade 3 or 4; five (2%) participants on the control regimen and seven (3%) on the oral regimen had died. Hearing loss (Brock grade 3 or 4) was more frequent in participants on the control regimen than in those on the oral regimen (18 [9%] *vs* four [2%], p=0·0015). Of 134 participants in the mITT population who were allocated to the 6-month regimen, 122 (91%) had a favourable outcome compared with 87 (69%) of 127 participants randomly assigned concurrently to the control regimen (adjusted difference 22·2%, 95% CI 13·1–31·2); six (4%) of 143 participants on the 6-month regimen had grade 3 or 4 hearing loss.

**Interpretation:**

Both bedaquiline-containing regimens, a 9-month oral regimen and a 6-month regimen with 8 weeks of second-line injectable, had superior efficacy compared with a 9-month injectable-containing regimen, with fewer cases of hearing loss.

**Funding:**

USAID and Janssen Research & Development.

## Introduction

Multidrug-resistant (MDR) or rifampicin-resistant tuberculosis is challenging to treat and, historically, outcomes have been poor. Until the past few years, very little evidence from randomised controlled trials was available to guide management. Globally, only one in three people who developed MDR or rifampicin-resistant tuberculosis in 2020 were started on treatment.^1^ Although outcomes are improving, only 59% of those starting treatment in 2018 completed it successfully. Effective, short, and well tolerated regimens that are easy to administer are urgently needed.

STREAM stage 1 was the first international phase 3 controlled clinical trial to evaluate a shortened treatment regimen for MDR or rifampicin-resistant tuberculosis. The trial showed that the efficacy of a 9-month regimen that included moxifloxacin and isoniazid at higher than standard dose, clofazimine and other drugs available at the time, and kanamycin in a 16-week intensive phase, was non-inferior to the 20-month regimen recommended by WHO between 2011 and 2018.^2^ At 132 weeks from randomisation, 78·8% of individuals allocated to the 9-month regimen had a favourable status, as did 79·8% of those allocated to the longer regimen.^3^ Despite the considerably reduced treatment duration, which generated substantial patient and health system economic benefits,^4^ participants on both regimens had a similar frequency of grade 3 or higher adverse events during treatment and follow-up.^3^

Before completion of STREAM stage 1, the trial was expanded to include a second stage to assess two new shortened treatment regimens containing bedaquiline, a drug that received accelerated regulatory approval by the US Food and Drug Administration in 2012; bedaquiline was the first new drug for tuberculosis discovered in over 40 years. The primary objective of STREAM stage 2 was to determine whether the proportion of participants with a favourable efficacy outcome at week 76 on a 9-month oral bedaquiline-containing regimen was non-inferior to the 9-month regimen assessed in STREAM stage 1 and, if non-inferiority was shown, to test for superiority. Assessment of a 6-month bedaquiline-containing regimen was a key secondary objective. A within-trial economic evaluation was also done and will be reported separately.

## Methods

### Study design

STREAM stage 2 was a randomised, phase 3, non-inferiority trial done in 13 hospital clinics in seven countries (Ethiopia, Georgia, India, Moldova, Mongolia, South Africa, and Uganda). The trial methods have been published;^3,5,6^ additional details are provided in the appendix (pp 7–19). Here, we describe the efficacy and safety outcomes from randomisation to 76 weeks; follow-up is continuing to 132 weeks. The Union Ethics Advisory Group was the global ethics committee. Ethical approvals were also obtained from national and institutional ethics committees of participating sites.

### Participants

Eligible participants were aged 15 years or older (where approved, otherwise 18 years or older) and had pulmonary tuberculosis (confirmed by positive sputum smear or nucleic acid amplification test [GeneXpert, Cepheid; Sunnyvale, CA, USA]) with evidence of resistance to rifampicin regardless of susceptibility to isoniazid. Participants were ineligible if they were infected with a strain of *Mycobacterium tuberculosis* resistant to a second-line injectable drug or fluoroquinolone (determined by line-probe assay); a complete list of inclusion and exclusion criteria is provided in the appendix (pp 10–11). Written informed consent was obtained from all participants.

### Randomisation and masking

Participants were randomly assigned 1:2:2:2 to one of four treatment regimens denoted as long regimen, control regimen, oral regimen, and 6-month regimen (details in the following subsection). Randomisations were stratified by site, HIV status, and CD4 count. Separate randomisation lists for each combination of strata were prepared by an independent statistician with permuted blocks of varying sizes. Participants were randomly assigned by site staff using a web-based randomisation system; if web access was not available at the time of randomisation, a manual alternative with use of sealed envelopes was provided. In 2018, the protocol was amended to close recruitment to the long and 6-month regimens.^4^ Randomisation to the 6-month regimen continued in India because cessation of recruitment to that treatment group was not approved locally. Participants and clinicians were aware of regimen assignments, but laboratory staff were not. Only the independent data monitoring committee and the unmasked statisticians saw aggregate data by treatment group during the trial.

### Procedures

**Figure 1:**
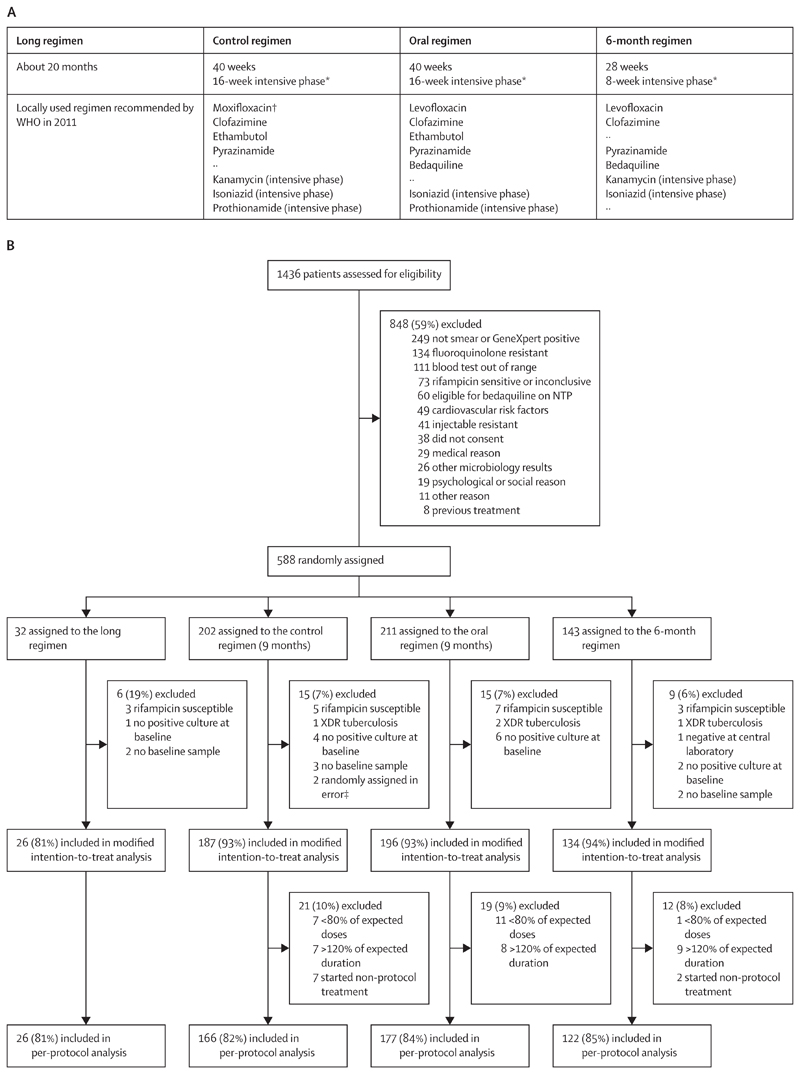
STREAM stage 2 regimen description (A) and flow diagram (B) Further information including dosing is given in the appendix (pp 8–10). NTP=national tuberculosis programme. XDR=extensively drug resistant. *The intensive phase should be extended by 4 or 8 weeks for patients whose smear has not converted. †Moxifloxacin was replaced by levofloxacin in 2018 due to the extent of QT prolongation seen in STREAM stage 1. ‡One patient had QTcF higher than 450 ms, and one patient had pre-existing hearing loss.

The trial regimens were the following: the long regimen was a 20-month regimen recommended by WHO from 2011 to 2018;^2^ the control regimen was a 9-month regimen comprised of moxifloxacin (at higher than standard dose), clofazimine, ethambutol, and pyrazinamide for 40 weeks, with kanamycin, high-dose isoniazid, and prothionamide given for the 16-week intensive phase; the oral regimen was a 9-month oral regimen identical to the control regimen, except that bedaquiline for 40 weeks replaced kanamycin and levofloxacin replaced moxifloxacin; and the 6-month regimen was a regimen lasting 6 months consisting of bedaquiline, clofazimine, pyrazinamide, and levofloxacin prescribed for 28 weeks, supplemented by high-dose isoniazid with kanamycin for an 8-week intensive phase (figure 1A). More details on the dose, route, and schedule of drug administration are presented in the appendix (pp 8–10). All regimens included the option to extend the intensive phase by up to 8 weeks for delayed sputum smear conversion. In 2018, a protocol amendment substituted levofloxacin for moxifloxacin in the control regimen when the results of STREAM stage 1 became known, with the aim of reducing the number of participants having QT prolongation.^5^ The medications in the long regimen were provided by the national tuberculosis programmes at their respective trial sites. Medications for the other regimens were procured by the trial sponsor (Vital Strategies; New York, NY, USA) from quality-assured sources, except for bedaquiline, which was provided by Janssen Research & Development (Raritan, NJ, USA).

Trial visits were weekly for the first 4 weeks, 4-weekly until week 52, then 8-weekly until week 76. Sputum samples for smear and culture were obtained at every visit from week 4 onwards. The trial reference laboratory tested *M tuberculosis* isolates for drug susceptibility from week 8 onwards and genotyped strains to distinguish true relapses from exogenous reinfections. Regular electrocardiogram monitoring with centralised review was done because of the potential for QT interval prolongation associated with fluoroquinolones, clofazimine, and bedaquiline. The corrected QT interval was calculated with use of Fridericia’s formula (QTcF) and treatment was modified if necessary to maintain QTcF <500 ms. Tablet-based audiometry testing and safety blood tests were done regularly during treatment and follow-up. A full list of the assessments done is provided in the appendix (pp 14–16).

### Outcomes

The primary efficacy outcome was favourable status at 76 weeks. This was defined as a negative culture for *M tuberculosis* at week 76 and on the preceding visit, with no intervening positive culture or previous unfavourable outcome. Unfavourable outcomes included the following: treatment initiation with bedaquiline, kanamycin, linezolid, or two or more other drugs if they were not part of the assigned regimen; treatment extension beyond the permitted duration; death from any cause; a positive culture from one of the two most recent specimens; or no week 76 visit.

Secondary efficacy outcomes were times to unfavourable outcome, probable or definite failure or recurrence (FoR),^7^ and smear and culture conversion; and frequency of acquired resistance to fluoroquinolones, aminoglycosides, bedaquiline, clofazimine, or pyrazinamide. Safety outcomes were the following: death from any cause; severe adverse events (grade 3 or higher according to the Division of AIDS, National Institute of Allergy and Infectious Diseases criteria^8^ except audiometry results, which were graded with Brock’s criteria^9^); and modification of treatment due to an adverse event. Additional safety outcomes were an analysis of serious adverse events, an analysis of QTcF interval prolongation, and changes in liver function and hearing loss. Only treatment-emergent adverse events are reported. We coded adverse events using the Medical Dictionary for Regulatory Activities (MedDRA, version 20.0). For the FoR analysis, an independent clinician, unaware of treatment-group assignment, reviewed data to the time of unfavourable efficacy outcome to determine the likelihood of failure or recurrence. An independent death review committee (two infectious disease specialists and a cardiologist), with members unaware of treatment-group assignments, classified the probable causes of death as cardiac-structural, cardiac-arrhythmic (ie, probable or possible sudden cardiac death), tuberculosis-related, HIV-related, or other. Exploratory analyses compared the oral and 6-month regimens in participants who were randomly assigned concurrently.

### Statistical analysis

We estimated that a sample of 200 participants allocated to each regimen would give 80% power to show the non-inferiority of the oral regimen versus the control regimen at a one-sided significance level of 0·025 using a 10% margin of non-inferiority. This assumed the following: a favourable efficacy outcome at 76 weeks in 80% of participants in the control regimen and 82% of those in the oral regimen, and 14% of participants excluded in the per-protocol analysis.

In the primary efficacy analysis, we calculated the absolute between-group difference (with 95% CI) in the proportion of participants with a favourable outcome, adjusted for HIV status and randomisation protocol, using Cochran-Mantel-Haenszel weights.^10^ Non-inferiority was shown if the upper boundary of the 95% CI was less than 10% in both the modified intention-to-treat (mITT) and per-protocol populations. The mITT population included all randomly assigned participants with a positive culture for *M tuberculosis* at screening or randomisation, except for participants with isolates taken before randomisation who were subsequently found to be susceptible to rifampicin or resistant to both fluoroquinolones and second-line injectables on phenotypic drug-susceptibility testing. The per-protocol population is the same as the mITT population with the exclusion of participants who did not complete a protocol-adherent course of treatment, other than for treatment failure, change of treatment for an adverse event, or death (appendix pp 16–17). We did one-sided tests for non-inferiority and calculations of the 95% CIs using the Wald standard error. Prespecified tests for superiority were done when non-inferiority was shown. The proportion of participants meeting each safety outcome was calculated similarly in the safety population, which comprised all participants who received at least one dose of a trial medication, with two-sided tests of superiority. We included a Bayesian interpretation of the primary outcome as a supplementary analysis. Prespecified sensitivity analyses of the primary efficacy analysis included analyses that were unadjusted, adjusted for important baseline characteristics, and where the definition of an unfavourable outcome was modified (appendix p 25). The primary efficacy analysis was also repeated in subgroups according to prespecified baseline characteristics (appendix p 26). We analysed time-to-event outcomes using the Kaplan-Meier product limit estimator, log-rank tests for differences between groups, and Cox-proportional hazards models; these were displayed with the KMunicate format.^11^ We tested assumptions of proportional hazards using Schoenfeld residuals.

All comparisons were restricted to participants randomly assigned concurrently. Analyses other than the primary outcome were stratified by randomisation protocol alone. Baseline characteristics and treatment adherence were summarised using counts and percentages or medians and IQRs. All analyses were done in STATA, version 17.0. An independent Trial Steering Committee oversaw the study with advice from an independent Data Monitoring Committee who regularly reviewed unblinded trial data. This trial is registered with ISRCTN, ISRCTN18148631.

### Role of the funding source

The funders of the study had no role in study design, data collection, data analysis, data interpretation, or writing of the report, except that Janssen Research & Development, as the developer of bedaquiline, provided a consultancy service upon request of the Sponsor in relation to bedaquiline, the eligibility criteria, safety investigations, and the pharmacokinetic component to fulfil the regulatory requirements of the trial.

## Results

Between March 28, 2016, and Jan 28, 2020, 1436 participants were screened and 588 were randomly assigned to the long regimen (32), control regimen (202), oral regimen (211), or 6-month regimen (143). Participants were recruited in Ethiopia (67), Georgia (32), India (148), Moldova (63), Mongolia (130), South Africa (92), and Uganda (56; appendix p 20). Reasons for exclusion from the analysis population are described in figure 1B. Few participants were randomly assigned to the long regimen due to early termination of recruitment; results for that regimen will be reported with the longer-term follow-up. Of the 588 participants randomly assigned, 517 were included in the mITT and 465 in the per-protocol analyses (appendix p 21).

Of the 517 participants in the mITT population, 320 (62%) were men and 197 (38%) were women, with a median age of 32·5 years (IQR 26·3–41·9). The demo-graphic characteristics of participants in the mITT population were broadly similar across regimens, although fewer participants in the control regimen had multiple cavities or had received previous treatment than in other regimens (table 1, appendix p 22). 73 (14%) of 517 participants in the mITT population were living with HIV, the majority from South Africa and Uganda; all except one were receiving combination anti-retroviral treatment at the time of enrolment or started within 8 weeks of enrolment.

The median duration of the allocated treatment was close to the intended length in all groups: 40·1 weeks (IQR 40·1–40·7) for the oral regimen, 40·1 weeks (40·1–40·3) for the control regimen, 28·1 weeks (28·1–28·1) for the 6-month regimen, and 40·1 weeks (40·1–40·3) for the concurrent control regimen. The intensive phase of treatment was extended because of delayed smear conversion in 20 (10%) of 196 participants on the oral regimen (13 by 4 weeks and seven by 8 weeks), and 16 (9%) of 187 on the control regimen (ten by 4 weeks and six by 8 weeks). Corresponding figures for the 6-month and control regimen comparison were seven (5%) of 134 participants on the 6-month regimen (five by 4 weeks and two by 8 weeks) and 13 (10%) of 127 on the control regimen (eight by 4 weeks and five by 8 weeks). Retention and self-reported adherence in all groups were good; at 76 weeks, 491 (95%) participants in the mITT population were seen or were known to have died (appendix p 23).

**Figure 2:**
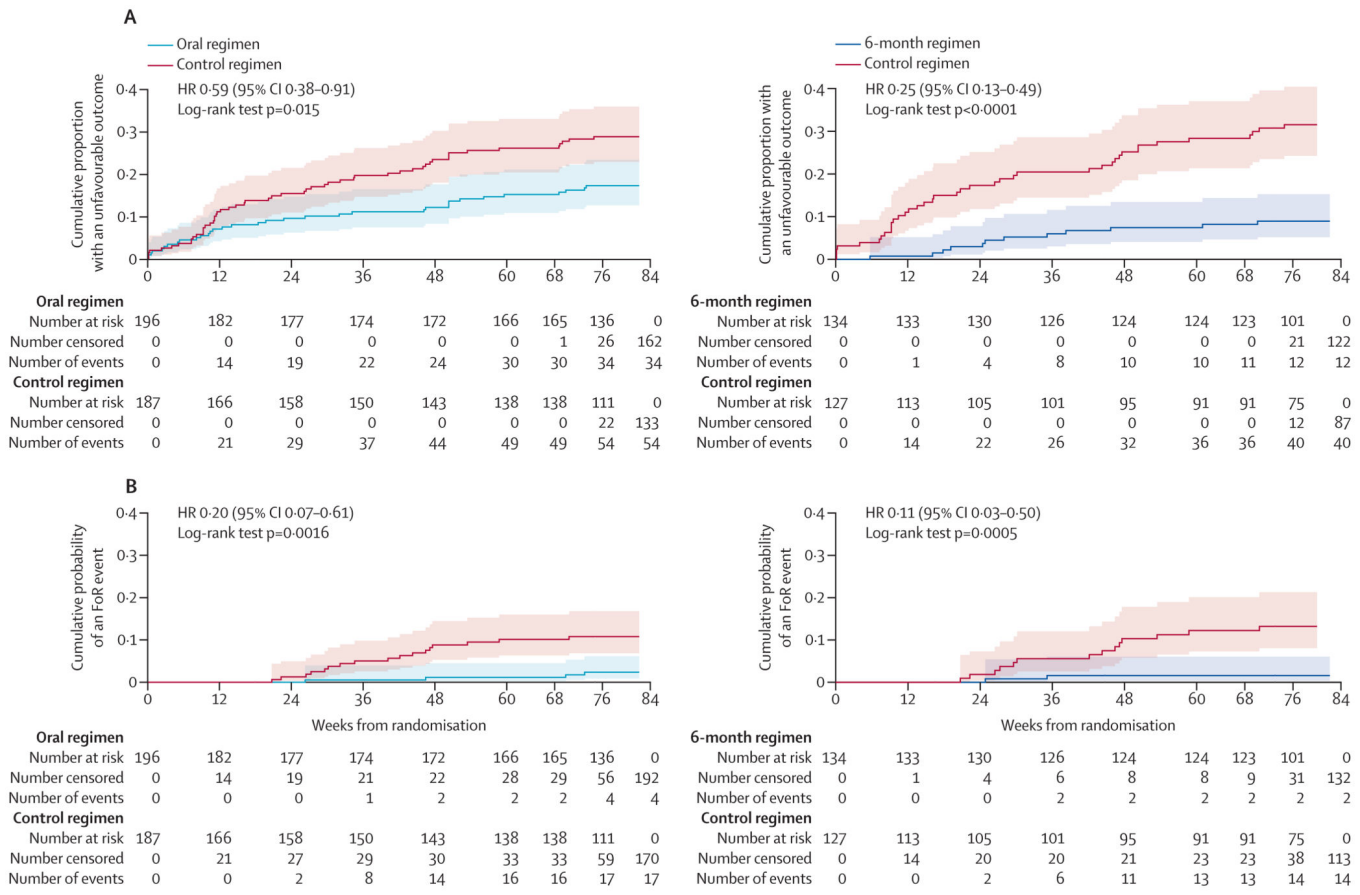
Time to unfavourable outcome (A) and failure or recurrence (B) HR=hazard ratio. FoR=failure or recurrence.

In the mITT analysis, 162 (83%) participants on the oral regimen achieved a favourable outcome compared with 133 (71%) on the control regimen (table 2), a difference of 11·0% (95% CI 2·9–19·0, p<0·0001) with a significant difference in time to an unfavourable outcome (figure 2A). In the per-protocol analysis, 155 (88%) participants on the oral regimen achieved a favourable outcome compared with 126 (76%) on the control regimen, a difference of 10·7% (2·9–18·5, p<0·0001; appendix p 24).

The proportion of participants in the mITT population whose unfavourable outcome was based on bacteriology was higher in the control (20 [37%]) than in the oral regimen (eight [24%]). No recurrence was identified as reinfection. Of 19 participants on the control regimen with drug-susceptibility testing before starting salvage treatment, one developed resistance to fluoroquinolones, one to clofazimine, one to pyrazinamide, and two to kanamycin; of seven tested on the oral regimen, one developed resistance to clofazimine, one to fluoroquinolones, one to fluoroquinolones and clofazimine, and one to bedaquiline and clofazimine (appendix p 25).

Treatment changes and extensions after adverse events were more frequent in the control than in the oral regimen, accounting for 24 (44%) unfavourable outcomes in the control regimen and nine (26%) in the oral regimen (table 2). Of 20 participants on the control regimen who changed treatment after an adverse event, six started bedaquiline and 14 started linezolid (including one who also started bedaquiline); the adverse events leading to treatment change were hearing disorders (16 participants), renal disorders (three), and injection site reaction (one). All six participants on the oral regimen who changed treatment due to adverse events had hepatic disorders and started kanamycin.

We did several sensitivity analyses of the mITT primary endpoint, including adjusting for baseline characteristics and variations in the definition of unfavourable outcome (appendix p 25). Non-inferiority of the oral regimen was shown in all these analyses.

The treatment effect (oral regimen *vs* control) did not differ significantly between the subgroups evaluated in the mITT analysis population, except in analyses by country and HIV status (appendix p 26). In Mongolia, the proportion of participants with a favourable outcome was higher in the control regimen (38 [84%] of 45 participants) than in the oral regimen (36 [78%] of 46 participants). Participants living with HIV were nearly all recruited from South Africa and Uganda; because most participants in those two countries combined were HIV positive, the effects of country and HIV infection cannot be easily separated. Participants living with HIV on the oral regimen had substantially better outcomes than participants on the control regimen; this difference was largely due to the control regimen performing less well in participants living with HIV (26 [96%] of 27 participants living with HIV had a favourable outcome on the oral regimen *vs* nine [36%] of 25 on the control regimen). The lower favourable outcome rate in participants living with HIV on the control regimen was due to a larger proportion of early changes to treatment for adverse events than in those without HIV infection (ten of 16 unfavourable outcomes in those living with HIV compared with ten of 38 in those without HIV infection on the control regimen). 136 (80%) of 169 participants without HIV infection on the oral regimen had a favourable outcome compared with 124 (77%) of 162 on the control regimen.

A favourable outcome was achieved in 87 (69%) of 127 participants on the control regimen with moxifloxacin compared with 103 (79%) of 131 on the oral regimen, a difference of 9·2% (95% CI –1·2 to 19·6). When levofloxacin was used in the control regimen, a favourable outcome was achieved in 46 (77%) of 60 participants on the control regimen compared with 59 (91%) of 65 participants on the oral regimen, a difference of 14·7% (2·5 to 26·8).

In the FoR analysis of tuberculosis-related outcomes, significantly fewer participants on the oral regimen had a definite or probable FoR event (p=0·0016, log-rank test, figure 2B): a Kaplan-Meier probability of a FoR event by week 76 of 0·11 (95% CI 0·07–0·17) for the control regimen and 0·02 (0·01–0·05) for the oral regimen. This difference remained in all sensitivity analyses (appendix p 27).

Median times to conversion to a negative smear and culture did not differ significantly between the treatment groups (appendix pp 28, 30).

The Bayesian interpretation of the results is presented in the appendix (p 32). The difference between the flat, sceptical, and expected priors was minimal. Using the sceptical prior, the probability that the proportion of participants with a favourable outcome in the oral regimen is at least 5% more than in the control regimen was 0·92, and the probability that the oral regimen has superior efficacy to the control regimen was 0·99. The Bayesian mean estimate of the risk difference was 10·9% (95% credible interval 2·7–19·1).

In the mITT analysis of the 6-month regimen versus control regimen comparison, 122 (91%) of 134 participants on the 6-month regimen had a favourable outcome compared with 87 (69%) of 127 on the control (table 2), a difference of 22·2% (95% CI 13·1–31·2, p<0·0001). We also observed a significant difference in time to an unfavourable outcome between the two regimens (figure 2A). In the per-protocol analysis, 114 (93%) of 122 participants on the 6-month regimen had a favourable outcome compared with 82 (75%) of 110 in the control regimen, a difference of 18·1% (9·3–27·0, p<0·0001).

The proportion of participants with an unfavourable outcome due to bacteriological reasons in the mITT analysis was higher in the control regimen (16 [40%] of 40) than in the 6-month regimen (three [25%] of 12; table 2). Of 15 participants on the control regimen with drugsusceptibility testing before starting salvage treatment, one developed resistance to clofazimine, one to fluoroquinolones, and one to pyrazinamide; of the three on the 6-month regimen, all three developed resistance to fluoroquinolones.

Treatment changes and extensions after adverse events were more common in the control than in the 6-month regimen, accounting for 17 (42%) unfavourable outcomes in the control regimen and four (33%) in the 6-month regimen. Of 14 participants on the control regimen who changed treatment after an adverse event, five started bedaquiline (all for hearing disorders), eight started linezolid (six for hearing disorders and two for renal disorders), and one started two or more other drugs (renal disorder). For the 6-month regimen, one participant started linezolid (QT prolongation) and two started two or more other drugs (both hepatic disorders).

In the FoR analysis, significantly fewer participants on the 6-month regimen had a definite or probable FoR event (p<0·0001, log-rank test, figure 2B), with a Kaplan-Meier probability of a FoR event by week 76 of 0·02 (95% CI 0·04–0·06) for the 6-month regimen and 0·13 (0·08–0·21) for the control regimen. The median times to conversion to a negative smear and culture did not differ significantly between regimens (appendix pp 29, 31).

14 participants, five (2%) allocated to the control regimen, seven (3%) allocated to the oral regimen, and two (1%) allocated to the 6-month regimen died during either treatment or follow-up. The small numbers of deaths and the variety of causes recorded (appendix p 33) make any pattern difficult to discern; there were two possible sudden cardiac deaths, one on the control and the second on the oral regimen, although all recorded QTcF measurements in both participants were shorter than 460 ms.

In the safety population (all randomly assigned participants), we observed no indication of a difference between the regimens in the proportion of participants who had a serious adverse event, a treatment-related serious adverse event, or a grade 3 or 4 adverse event (table 3, appendix pp 33–40).

A grade 3 or 4 adverse event or death occurred in 109 (54%) of 202 participants allocated to the control regimen compared with 109 (52%) of 211 allocated to the oral regimen and 81 (57%) of 143 allocated to the 6-month regimen. The most common category of grade 3 or 4 adverse event was the “Torsade de pointes–QT prolongation” standardised MedDRA query, identified in approximately a quarter of participants on all three regimens (appendix pp 41–50). No cases of torsade were reported; most events were QTcF increases from baseline of 60 ms or higher; QTcF reached 500 ms or higher in only a small proportion of participants (table 3).

We observed similar patterns of mean QTcF changes in the oral and control regimens, reaching a plateau of an approximately 30 ms increase at week 16, which declined at the end of treatment (appendix p 52). On the 6-month regimen, the increase appeared to be slightly greater than in the control regimen, but it declined rapidly after the end of treatment at 28 weeks.

The second most commonly reported category of grade 3 or 4 adverse events was hepatic disorders, both during allocated treatment and at any time to 76 weeks (appendix pp 41–50). During allocated treatment, 20 (10%) participants on the control regimen and 26 (12%) of those on the oral regimen had a severe hepatic event; these were slightly less frequent on the 6-month regimen, reported in seven (5%) participants. However, the proportion of participants with either aspartate aminotransferase or alanine aminotransferase more than five times the upper limit of normal, or with aspartate aminotransferase three times the upper limit of normal combined with bilirubin more than twice the upper limit of normal, were similar in all regimens (table 3).

Treatment-emergent hearing loss graded 3 or 4 on the Brock scale, indicating sensorineural hearing loss,^9^ was recorded in significantly more participants on the control regimen than those on the oral regimen (18 [9%] *vs* four [2%], p=0·0015). Fewer participants allocated to the 6-month regimen had Brock grade 3 or 4 hearing loss than those allocated to the control regimen (six [4%] *vs* 11 [8%], p=0·20).

A higher proportion of participants on the control regimen than on the oral or 6-month regimens required a permanent discontinuation of a drug after an adverse event (37 [18%] on the control regimen *vs* 15 [7%] on the oral regimen and eight [6%] on the 6-month regimen; appendix p 51). On the control regimen, the most common reason for drug discontinuation was for hearing and vestibular disorders. On all three regimens, we observed similar small numbers of drug discontinuations for gastrointestinal disorders, hepatic disorders, and QT prolongation.

In exploratory analyses comparing the 6-month regimen with the oral regimen, a significantly higher proportion of participants on the 6-month regimen had a favourable outcome (122 [91%] *vs* 103 [79%]), a difference of 12·5% (95% CI 4·2–20·8, p=0·0016). We observed no significant difference in the proportion of participants who had a grade 3 or 4 adverse event (65 [45%] on the oral regimen *vs* 79 [55%] on the 6-month regimen, p=0·086). Two deaths (1%) were recorded in each regimen.

## Discussion

This study provides robust evidence that, 76 weeks from randomisation, two bedaquiline-containing regimens—an oral 9-month regimen and a 6-month regimen including 8 weeks of a second-line injectable—were not only non-inferior, but superior in efficacy to the 9-month control regimen in participants with rifampicin-resistant tuberculosis without evidence of resistance to fluoroquinolones or aminoglycosides on line probe assay. The control regimen was evaluated in STREAM stage 1 and recommended by WHO when STREAM stage 2 began in 2016,^12^ a recommendation that was superseded in 2020 when, because of concerns about hearing loss associated with aminoglycosides, WHO endorsed a 9-month, bedaquiline-containing, injectable-free alternative used in the South African national tuberculosis treatment programme.^13^ Therefore, the results of STREAM stage 2 should be considered in the context of both the control regimen and the available evidence regarding efficacy and safety of regimens currently recommended by WHO.

STREAM stage 2 makes an important contribution to the growing body of evidence available to support treatment guidelines for MDR and rifampicin-resistant tuberculosis. In addition to the efficacy and safety of the regimens, the health economic component of STREAM stage 2 provides evidence on the probable costs of their implementation from both provider and patient perspectives. Current WHO recommendations^13^ are based largely on single-country and unpublished trial results, supplemented by observational data. The superiority of the oral regimen compared with the control regimen validates WHO’s current recommendation of a 9-month, bedaquiline-based oral regimen, which was based only on observational data.^13,14^

In May, 2022, WHO announced that, in its forthcoming guidelines, it would also be recommending programmatic use of a 6-month bedaquiline, pretomanid, and linezolid-based regimen in people with MDR or rifampicin-resistant tuberculosis or pre-extensively drug-resistant tuberculosis, as a shorter alternative to a 9-month regimen.^14^ This recommendation was based on the findings of Nix-TB, a single-arm study in participants with extensively drug-resistant tuberculosis or treatment-intolerant or non-responsive MDR tuberculosis,^15^ and the unpublished results of the TB-PRACTECAL^16^ and ZeNix trials (subsequently published^17^).

The results of NExT^18^—a South African randomised trial of a 6-month oral five-drug regimen that includes bedaquiline, linezolid, and levofloxacin—also support the use of a 6-month regimen. Differences in outcome definition make direct comparisons difficult, which could be remedied by harmonisation of trial endpoints.

In addition to the WHO-recommended regimens outlined, STREAM provides information on an effective 6-month alternative, which could be useful in some settings, particularly where there are concerns about linezolid toxicity. Even at the 600 mg dose used in ZeNix, linezolid can cause peripheral neuropathy and myelosuppression.^19^ In ZeNix, 24% of participants allocated to linezolid 600 mg for 26 weeks had an episode of peripheral neuropathy, although most of these were grade 1; this risk would need to be compared with the risk of ototoxicity associated with 8 weeks of a second-line injectable in the STREAM 6-month regimen.

The primary efficacy endpoint of STREAM was a composite outcome, including both bacteriological unfavourable events (failure, reversion, or reinfection) and non-bacteriological unfavourable events, such as deaths from any cause and major changes to the allocated regimen for toxicity or other reasons. Although the non-bacteriological causes predominated in all three regimens, both bacteriological and non-bacteriological unfavourable outcomes were more frequent in the control regimen than in either of the bedaquiline-containing regimens, suggesting that the superiority of the bedaquiline-containing regimens was not simply due to better tolerability. The secondary FoR analysis showed that there was a very low risk of failure or recurrence in both intervention regimens when compared with the control regimen; we also found no evidence to suggest that participants with more extensive disease fared worse than those with limited disease. The number of participants with acquired resistance to any of the key drugs based on phenotypic drug-susceptibility testing was small in all regimens. Acquired resistance to bedaquiline is of particular concern, but only one case was reported in our study.

Outcomes in participants with rifampicin-resistant tuberculosis and HIV co-infection are important because of the close association of the two infections, particularly in sub-Saharan Africa, and the challenges they pose for both patients and health systems. Outcomes were considerably worse in the control regimen than they had been when that regimen was given in STREAM stage 1; in STREAM stage 2, a high proportion of the unfavourable outcomes in participants living with HIV were treatment changes after an adverse event occurring early in treatment. It is probable that the increased availability of alternative treatments contributed to the difference.

The frequencies of the main safety parameters during treatment and follow-up, namely deaths, grade 3 or 4 adverse events, serious adverse events, and treatment-related serious adverse events, were similar on all three regimens, suggesting that overall no regimen was better or worse from a safety perspective than the others. Although over half the participants were reported to have had a grade 3 or 4 adverse event, many of these were identified from the frequent laboratory and other investigations undertaken and were not necessarily associated with clinical disease. Of note, very few serious adverse events were considered by the treating physician to be treatment related.

The results of the audiometry monitoring are particularly important. Site investigators used the results for patient management when any grade of impairment was noted but, despite treatment modification and early discontinuation of kanamycin in participants affected, the control regimen had significantly higher rates of hearing loss than the oral regimen. The observation that Brock grade 3 or higher hearing loss was halved in the 6-month regimen compared with the control regimen suggests that the reduced aminoglycoside exposure successfully reduced ototoxicity but did not eliminate it.

Four of the drugs used in STREAM stage 2 are associated with QT prolongation—moxifloxacin, levofloxacin, clofazimine, and bedaquiline—and all the regimens studied included at least two of these drugs. QT prolongation was common in all three regimens; however, in only a small proportion of participants (3–6%) did the QTcF interval reach 500 ms or higher, the threshold at which the risk of serious arrhythmia starts to increase,20 requiring treatment modification.

Few deaths occurred, with no clear pattern. The independent review identified two possible sudden cardiac deaths but with no evidence of QT prolongation, one each on the control and oral regimens. Because of concerns due to possible increased mortality in the bedaquiline-containing group seen after the end of treatment in the C208 trial,^21^ long-term safety follow-up to week 132 is ongoing. As STREAM stage 2 is the first trial to study a treatment regimen with more than 6 months of bedaquiline, this will have important clinical implications.

An exploratory analysis of the comparative efficacy of the 6-month and oral regimens showed significantly better efficacy of the 6-month regimen compared with the oral regimen. Whether this could outweigh the attendant small increased risk of hearing loss and whether it would be acceptable to patients and health systems requires further consideration. It is possible that an even shorter period of second-line injectable would be beneficial and would be worth investigating.

The key strengths of the trial were the diversity of the population (sites in seven countries on three continents, with different ethnic compositions and health-care systems), the inclusion of participants co-infected with HIV, and a greater than 90% rate of retention.

The main limitation of the trial is that the open-label design might have influenced decisions on regimen change, especially for non-bacteriological reasons. However, the number of treatment changes that were not related to toxicity were few and occurred with similar frequency across the treatment groups.

In conclusion, STREAM stage 2 has shown that two short-course, bedaquiline-containing regimens are not only non-inferior but superior to a 9-month injectable-containing regimen. The STREAM stage 2 fully oral regimen avoided the toxicity of aminoglycosides, and the 6-month regimen was highly effective, with reduced levels of ototoxicity. These two regimens offer promising treatment options for patients with MDR or rifampicin-resistant tuberculosis. However, safer and simpler alternatives are still needed.

## Supplementary Material

Supplementary

## Figures and Tables

**Table 1:** Baseline characteristics of modified intention-to-treat analysis population

	Oral regimen *vs* control regimen		6-month regimen *vs* control regimen	
	Control	Oral	Control	6-month
Total in mITT population	187	196	127	134
Gender				
Men	115(61%)	124 (63%)	77 (61%)	81 (60%)
Women	72 (39%)	72 (37%)	50 (39%)	53 (40%)
Age, years				
<25	44 (24%)	33 (17%)	31 (24%)	32 (24%)
25–44	105 (56%)	119 (61%)	73 (57%)	79 (59%)
≥45	38 (20%)	44 (22%)	23 (18%)	23 (17%)
Weight, kg				
<33	1 (1%)	2 (1%)	1 (1%)	3 (2%)
33–50	64 (34%)	86 (44%)	50 (39%)	55 (41%)
≥50	122 (65%)	108 (55%)	76 (60%)	76 (57%)
Body-mass index, kg/m^2^				
<16·0	21 (11%)	29 (15%)	14 (11%)	23 (17%)
16·0–18·4	52 (28%)	60 (31%)	38 (30%)	31 (23%)
18·5–24·9	96 (51%)	92 (47%)	66 (52%)	72 (54%)
≥25·0	18 (10%)	15 (8%)	9 (7%)	8 (6%)
HIV status and CD4 count, cells/mm^3^			
Negative	162 (87%)	169 (86%)	106 (83%)	113 (84%)
50–349	12 (6%)	13 (7%)	10 (8%)	10 (7%)
≥350	13 (7%)	14 (7%)	11 (9%)	11 (8%)
Smoking status				
Never smoked	119 (64%)	114 (58%)	87 (69%)	96 (72%)
Ex-smoker	40 (21%)	51 (26%)	18 (14%)	22 (16%)
Current smoker	28 (15%)	31 (16%)	22 (17%)	16 (12%)
Previous tuberculosis treatment			
None	60 (32%)	40 (20%)	33 (26%)	26 (19%)
Drug-sensitive tuberculosis	65 (35%)	93 (47%)	49 (39%)	55 (41%)
Second-line	62 (33%)	63 (32%)	45 (35%)	53 (40%)
Radiographic extent of disease*				
None or minimal	23 (13%; n=176)	13 (7%; n=184)	15 (13%; n=117)	12 (10%; n=124)
Moderate	100 (57%; n=176)	103 (56%; n=184)	65 (56%; n=117)	66 (53%; n=124)
Advanced	53 (30%; n=176)	68 (37%; n=184)	37 (32%; n=117)	46 (37%; n=124)
Unavailable or unassessable	11	12	10	10
Radiographic extent of cavitation*			
None	48 (27%; n=176)	45 (24%; n=184)	29 (25%; n=117)	31 (25%; n=124)
Single cavity	46 (26%; n=176)	24 (13%; n=184)	34 (29%; n=117)	22 (18%; n=124)
Multiple cavities	82 (47%; n=176)	115 (63%; n=184)	54 (46%; n=117)	71 (57%; n=124)
Unavailable or unassessable	11	12	10	10

Data are n (%) or n (%; N). mITT=modified intention-to-treat.

* Percentages are of non-missing values.

**Table 2:** Primary efficacy analysis in modified intention-to-treat population

	Oral regimen *vs* control regimen			6-month regimen *vs* control regimen		
	Control	Oral	Difference in favourable response*	Control	6-month	Difference in favourable response*
Total in mITT population	187	196	··	127	134	··
Total with a favourable outcome	133 (71%)	162 (83%)	11·0% (95% CI 2·9-19·0)	87 (69%)	122 (91%)	22·2% (95% CI 13·1-31·2)
Total with an unfavourable outcome	54 (29%)	34 (17%)	··	40 (31%)	12 (9%)	··
Unfavourable outcomes based on bacteriology						
Never achieved culture conversion†	6	2	··	5	1	··
Bacteriological reversion on treatment	11	3	··	8	1	··
Bacteriological recurrence after treatment‡	1	2	··	1	1	··
Culture positive at week 76	2	1	··	2	0	··
Unfavourable outcomes not based on bacteriology						
Died during treatment or follow-up (culture converted)	1	3	··	0	2	··
Lost to follow-up (culture converted)	3	6	··	2	2	··
Treatment changed after adverse event	20	6	··	14	3	··
Treatment extended after adverse event	4	3	··	3	1	··
Treatment extended or changed for other reasons	3	3	··	2	1	··
Participant withdrew consent	3	5	··	3	0	··

Data are n (%), unless otherwise stated. Table presents unfavourable outcomes that led to the primary endpoint, that is, the first unfavourable event that was classified as unfavourable for each participant. mITT=modified intention-to-treat.

* Analyses adjusted for randomisation protocol and HIV status.

† Includes three early deaths (one in control, two in oral).

‡ Includes one patient on the oral regimen who developed an empyema.

**Table 3:** Summary of safety outcomes

	Oral regimen *vs* control regimen		6-month regimen *vs* control regimen	
	Control	Oral	Control	6-month
Total in the safety analysis population	202	211	140	143
Participants with an SAE	35 (17%)	38 (18%)	26 (19%)	27 (19%)
Participants with treatment-related SAE	7 (3%)	4 (2%)	6 (4%)	6 (4%)
Death from any cause	5 (2%)	7 (3%)	2 (1%)	2 (1%)
Any grade 3-4 adverse event	108 (53%)	106 (50%)	75 (54%)	79 (55%)
Any grade 3-5 adverse event	109 (54%)	109 (52%)	76 (54%)	81 (57%)
QTcF >500 ms	12 (6%)	7 (3%)	8 (6%)	4 (3%)
ALT or AST >5-times ULN	28 (14%)	32 (15%)	15 (11%)	13 (9%)
ALT >3-times ULN and total bilirubin >2-times ULN	9 (4%)	14 (7%)	5 (4%)	7 (5%)
Brock grading ≥3 (either ear)	18 (9%)	4 (2%)	11 (8%)	6 (4%)

Data are n (%). ALT=alanine aminotransferase. AST=aspartate aminotransferase. SAE=serious adverse event. QTcF=corrected QT interval calculated with Fridericia’s formula. ULN=upper limit of normal.

## Research in context

**Evidence before this study**

Few randomised phase 3 clinical trials in participants with rifampicin-resistant tuberculosis have been completed and published. We searched PubMed for randomised treatment trials with clinical outcomes in rifampicin-resistant or multidrug-resistant tuberculosis, published from Jan 1, 2000, to April 22, 2022. We used the following search terms: “trial” AND “tuberculosis” AND “rifampicin resistance” OR “MDR” OR “multi-drug” OR “multidrug” OR “rifampicin-resistance”, with no language restrictions. This search yielded 243 results; studies that were not randomised control trials reporting clinical outcomes were excluded, leaving only four trials.

Our study was designed in 2016, before publication of the STREAM stage 1 results, which found the study regimen (the control regimen in STREAM stage 2) to be non-inferior to the treatment based on 2011 WHO guidelines. The only randomised clinical trial evidence at that time was the C208 study (2014), a phase 2b trial comparing bedaquiline or placebo added to an optimised background regimen. In addition to showing that bedaquiline reduced the median time to culture conversion (the primary outcome), cure rates at 120 weeks using WHO outcome definitions were 58% compared with 32% with placebo.

In 2019, a trial comparing delamanid or placebo added to an optimised background regimen was published. The primary endpoint showed no difference in sputum culture conversion, and neither was there any difference in the long-term outcome. In 2022, the NExT trial found that 51% of participants assigned to a 6-month all-oral regimen composed of WHO group A drugs plus two other group B or C drugs had a favourable outcome at 24 months compared with 23% assigned the injectable-based standard of care.

**Added value of this study**

The STREAM stage 2 study shows that both a 9-month oral bedaquiline-containing regimen and a 6-month bedaquiline-containing regimen including 8 weeks of a second-line injectable had superior favourable outcomes compared with a 9-month injectable-based regimen, with very little acquisition of phenotypic resistance to core drugs.

**Implications of all the available evidence**

The findings of the STREAM stage 2 trial, combined with results of previous trials, show that shorter bedaquiline-containing regimens are an effective treatment for patients with multidrug-resistant-tuberculosis.

## Data sharing

Data collected for the study, including individual participant data and a data dictionary defining each field in the set, will be made available no later than 12 months after the end of the trial through the TBPACT data repository (https://c-path.org/programs/tb-pacts/). We will provide de-identified participant data, data dictionary, study protocol, a set of blank case record forms, and the informed consent form.
