# Supplementary material for: Evaluation of two short standardised regimens for the treatment of rifampicin-resistant tuberculosis (STREAM stage 2): an open-label, multicentre, randomised, non-inferiority trial
Source: Lancet. Author manuscript; Available in PMC 2023 Jul 27. (PMC7614824; doi:10.1016/S0140-6736(22)02078-5)
Supplement: Supplementary [file EMS176871-supplement-Supplementary.pdf]

THE LANCET

Supplementary appendix

This appendix formed part of the original submission and has been peer reviewed. We post it as supplied by the authors.

Supplement to: Goodall RL, Meredith SK, Nunn AJ, et al. Evaluation of two short standardised regimens for the treatment of rifampicin-resistant tuberculosis (STREAM stage 2): an open-label, multicentre, randomised, non-inferiority trial. *Lancet* 2022; published online Nov 8. https://doi.org/10.1016/S0140-6736(22)02078-5.

Online Supplement

Contents

1 STREAM Study Collaborators and additional acknowledgements 3
1.1 STREAM Collaborators 3
1.2 Additional Acknowledgements 4
2 Background 5
3 STREAM Stage 2 objectives 6
3.1 STREAM Stage 2 primary objective: 6
3.2 STREAM Stage 2 secondary objectives: 6
4 Detailed Methods 7
4.1 Randomisation 7
4.2 Interventions 8
4.3 Participant full eligibility criteria 10
4.3.1 Inclusion criteria 10
4.3.2 Exclusion criteria 10
4.4 Study procedures 11
4.5 Bacteriological procedures 12
4.6 Assessment Schedule 14
4.7 Analysis definitions 16
4.7.1 Analysis Populations 16
4.7.2 Protocol adherent treatment 17
4.7.3 Primary efficacy outcome 17
4.7.5 Analysis of culture results 19
5 Supplementary Tables and Figures 20
Table S1: Summary of participants screened and randomised by centre 20
Table S2: Summary of randomisation allocation by country, mITT population 20
Table S3: Summary of Analysis Populations by treatment arm 21
Table S4: Additional baseline characteristics of the mITT population 22
Table S5: Retention at week 76 23
Table S6: Adherence by treatment arm in mITT population 23
Table S7: Primary outcome, per-protocol population 24
Table S8: Acquired resistance on allocated treatment 25
Table S9 Difference in proportion with a favourable outcome: Sensitivity analyses for the primary outcome 25

Figure S1 Difference in proportion with a favourable outcome: Subgroup analyses of the primary outcome 26

Table S10 Sensitivity analyses to account for informative censoring in time to FoR event 27

Table S11 Predictors of probability of censoring used in sensitivity analyses of time to FoR event 27

Table S12: Time to smear conversion: median survival time – Control vs Oral 28

Figure S2: Time to smear conversion: Kaplan-Meier survival estimates and hazard ratio – Control vs Oral 28

Table S13: Time to smear conversion: median survival time – Control vs Six-month 29

Figure S3: Time to smear conversion: Kaplan-Meier survival estimates and hazard ratio – Control vs Six-month 29

Table S14: Time to culture conversion: median surival time – Control vs Oral 30

Figure S4: Time to culture conversion: Kaplan-Meier survival estimates and Hazard ratio – Control vs Oral 30

Table S15: Time to culture conversion: median survival time – Control vs. Six-month 31

Figure S5: Time to culture conversion: Kaplan-Meier survival estimates and Hazard ratio – Control vs. Six-month 31

Figure S6 Flat, Sceptical and Expected priors for secondary Bayesian analysis. 32

Figure S7 Results of Bayesian analysis of non-inferiority 32

Table S16: Probable cause of death: results of Independent Death Review 33

Table S17: Grade 3-4 AE up to week 76: difference in proportion of participants with at least one event between treatment arms 33

Table S18: Grade 3-4 AE on allocated treatment: difference in proportion of participants with at least one event between treatment arms 33

Table S19: Summary of SAEs by System Organ Class and Preferred Term, up to week 76 34

Table S20: Summary of SAEs by System Organ Class and Preferred Term, time on allocated treatment only 38

Table S21: Summary of Grade 3-4 AEs by System Organ Class and Preferred Term, up to 76 weeks 41

Table S22: Summary of Grade 3-4 AEs by System Organ Class and Preferred Term, time on allocated treatment only 46

Table S23: Proportions of participants with change in trial regimen following AE 51

Table S24: Changes in regimen following AE, by allocated regimen 51

Figure S8: Plot of change from baseline in mean QT interval (ms) by treatment arm, over time from randomisation 52

Figure S9: Plot of change from baseline in mean QTcF interval (ms) by treatment arm, over time from randomisation 52

1 STREAM Study Collaborators and additional acknowledgements

1.1 STREAM Collaborators

Armauer Hansen Research Institute (AHRI), Addis Ababa, Ethiopia
Mekonnen Teferi and Helen Teklu

B.J. Medical College, Ahmedabad, India
Ghanshyam Borisagar

Clinical HIV Research Unit, Helen Joseph Hospital, University of the Witwatersrand, Johannesburg, South Africa
Jaclyn Bennet and Thando Mwelase

Institute of Phthisiopneumology "Chiril Draganiuc", Chisinau, Republic of Moldova
Sofia Alexandru and Irina Pirlog

Institute of Tropical Medicine, Antwerp, Belgium
Bouke de Jong and Leen Rigouts

King Dinuzulu Hospital Complex, Durban, South Africa
Nonhlanhla Gahima and Lisa White

Liverpool School of Tropical Medicine, Liverpool, UK
Laura Rosu

Makerere University Lung Institute, Mulago Hospital, Kampala, Uganda
Ivan Kimuli and Joanitah Nalunjogi

MRC CTU at UCL, UCL, London, UK
Katharine Bellenger, Deborah Bennet, Claire Cook, Andrew Davis, Wendy Dodds, Gareth Hughes, Brendan Murphy, Mary Rauchenberger, Carol Roach and Johanna Whitney

National Center for Communicable Diseases, Ulaanbaatar, Mongolia
Oyunchimeg Adilaa, Doljinsuren Dalai, Narangarav Tsegeen and Chuluunbaatar Zagd

National Center for Tuberculosis and Lung Diseases, Tbilisi, Georgia
Nino Lomtadze

National Institute for Research in Tuberculosis, Chennai, India
Thirumaran Senguttuvan, Rathinam Sridhar and Vignes Srinivasulu

Rajan Babu Institute for Pulmonary Medicine & Tuberculosis (RBIPMT), Delhi, India
Priyanka Bindroo and Mahmud Hanifa

REDE-TB (Brazilian TB Research Network)
Ezio Santos-Filho

St Peter's Tuberculosis Specialized Hospital and Global Health Committee, Addis Ababa, Ethiopia
Belay Gebreegziabher, Anne Goldfeld and Million Sisay

Think Tuberculosis & HIV Investigative Network, Doris Goodwin Hospital, South Africa
Lynette Duckworth and Odette van Amsterdam

Vital Strategies, New York, USA
Jan Komrska, Ishmael Qawiy, Leena Patel

1.2 Additional Acknowledgements

The STREAM study investigators and collaborators would like to thank the following people for their invaluable assistance with the conduct of the STREAM Stage 2 study:

Independent Trial Steering Committee
Prof. Robert Horsburgh [chair], Ms. Thandie Balfour, Prof.Frank Cobelens, Dr. Alwyn Mwinga, and Prof. Jae-Joon Yim

Independent Data Monitoring Committee
Prof. James Neaton [chair], Dr. Marta Boffito, Prof. Janet Darbyshire, Dr. Colin Forfar, Dr Maricelle Gler and Dr. Nesri Padayatchi

Independent Death Review Committee
Dr Yaver Bashir, Dr. Michael Brown and Dr. Robert Miller

Independent FoR Endpoint Review Committee
Prof. Marc Lipman

Armauer Hansen Research Institute (AHRI), Addis Ababa, Ethiopia
Netsanet Aragaw, Daniel Achalu and Rediet Fikru

B.J. Medical College, Ahmedabad, India
Rakesh Joshi

Clinical HIV Research Unit, Helen Joseph Hospital, University of the Witwatersrand, Johannesburg, South Africa
Nokuphiwa Mvuna and Mirriam Manamathela

Institute of Phthisiopneumology "Chiril Draganiuc", Chisinau, Republic of Moldova
Anna Donica, Svetlana Gheorghilas, Dumitru Popov, Olga Alexei, Silvia Macari, Valeriu Crudu, Nelly Ciobanu, Zinaida Corloteanu, Maria Gasco, Anghelina Djugostran, Iurie Vladei, Evghenia Cula, Irina Calancea, Domnita Jardan and Svetlana Doltu

Institute of Tropical Medicine, Antwerp, Belgium
Bart Derkinderen, Christel Desmaretz, Sara Sengstake, Kristien Van Camp

King Dinuzulu Hospital Complex, Durban, South Africa
Shakira Rajaram, Londiwe Luthuli, Sikhumbuzo Majola, Ella Lesego Ndlovu,Thobile Shinga, Nompumelelo Ndlovu, Zanele Dlokweni, Ravi Maharaj, Muziwandile Hezekial Ndlovu, Onke Hubela, Silindokuhle Goge, Deborah Chili and Nompumelelo Motaung

Makerere University Lung Institute, Mulago Hospital, Kampala, Uganda
Sharon Namiiro, Flavia Ojambo, Okello Ignatius, Muhwezi Kenneth, Sarah Onaga, Segawa Ivan, Susan Adakun, Winceslaus Katagira, Harriet Kisembo, Willy Ssengooba, Pheona Nsubuga, Moses Joloba and Paul Mbavu

MRC CTU at UCL, UCL, London, UK
Saiam Ahmed, Rachel Bennett, Angela Crook, Hanif Esmail, Cheryl Pugh, Ben Spittle and Conor Tweed

National Center for Communicable Diseases, Ulaanbaatar, Mongolia
Naranbat Nyamdawaa, Ganzorig Munkhjargal, Erdenesuvd Radnaabazar, Gantumur Ulziitumur, Bujintuul Ulziikhutag, Oyuntsetseg Sodnomdarjaa, Byambadulam Erdenekhuu, Bulgantsetseg Batkhuu, Gantulga Sanjaa, Bolortuya Adyabaatar, Tugsuu Sengedamba, Buyankhishig Burenbaatar, Baasansuren Erkhembayar, Budsuren Oyunbaatar, Zoljargal Avarzed, Bayasgalan Banzai, Amarjargal Norov, Erdenetsetseg Chuluunbaatar, Anarzul Bold and Ariunbold Lkhamsuren

National Center for Tuberculosis and Lung Diseases, Tbilisi, Georgia
Maia Kipiani and Mariana Buziashvili

National Institute for Research in Tuberculosis, Chennai, India
Ramesh Paranji Murugesan, Arun Babu Velmurugan, Balaji Subramanium, Menon Pradeep Aravindan, Srinivasa Basavewowdanadoddi Marinaik, Gomathi Narayan Sivaramakrishnan, Michael Premkumar, Guruprasad Sonuguru, Bhanu Kesavamurthy, Dharmarajan Panneerselvam, Vasuki Ranganathan, Lavanya Jayabal, Lakshmi Murali, Asha Fredricks, Rajasekharan Subramani, Keerthana Jyotheeswara Pillai, Dhanalakshmi Rajendran and Padmapriyadarsini Chandrasekaran

Rajan Babu Institute for Pulmonary Medicine & Tuberculosis (RBIPMT), Delhi, India
Pritti Gupta

St Peter's Tuberculosis Specialized Hospital and Global Health Committee, Addis Ababa, Ethiopia
Tolera Gerbaba, Adugna Bezabih, Yohannes Hailemichael, Birega Woldetsadik, Mohammad Yesuf, Mohammed Abseno, Asamenech Mola, Chalchisa Mulisa, Kidist Tsigehana, Rabia Gashaw, Selamawit Hagos, Rocio Hurtado andTamiru Assefa.

Think Tuberculosis & HIV Investigative Network, Doris Goodwin Hospital, South Africa
Louisa Dunn, Anita Jacobs, Hlengiwe Zondi, Manchali Zwane, Nokuphila Shoba, Mthokozisi Khuzwayo, Lungile Phakathi and Magic Khanyile

USAID
Dr YaDiul Mukadi

Vital Strategies, New York, USA
Kate West

2 Background

STREAM Stage 2 started as a four-arm randomised controlled trial based on a protocol that was a substantial amendment of the Stage 1 two-arm trial.

Vital Strategies, formerly known as the International Union Against Tuberculosis and Lung Disease Inc., is the sponsor of both Stage 1 and Stage 2 of STREAM. STREAM Stage 2 is the registration trial for bedaquiline and is regulated by the US FDA and the European Medicines Agency (EMA). It was approved by the Union's Ethics Advisory Group and the national and local ethics committees of all participating countries. The trial was conducted in Ethiopia (2 sites), Georgia (1 site), India (3 sites), Moldova (1 site), Mongolia (1 site), South Africa (4 sites) and Uganda (1 site).

A trial steering committee (TSC) with an independent chair supervised the conduct of the trial. An independent data monitoring committee (IDMC) met approximately every six months to oversee the safety of the study participants. Only the IDMC and the unblinded statisticians saw aggregate data by treatment arm during the trial. At each meeting of the IDMC they reviewed the accumulated safety and efficacy data by study arm. The IDMC was tasked with advising the TSC that the trial or a treatment arm should be stopped

if in their view there was unacceptable levels of drug toxicity or mortalilty or, there was proof beyond reasonable doubt from other studies to influence clinic staff in their management of patients that was incompatible with continuing the trial. The trial was not to be modified on account of differences in efficacy between treatment arms unless there was a concern for patient safety. They could also recommend modification or closure of the study in a country or sub-group of participants, such as those who are HIV-infected.

3 STREAM Stage 2 objectives

3.1 STREAM Stage 2 primary objective:

The primary objective of the Stage 2 comparisons of the STREAM trial is:

1. To assess whether the proportion of participants with a favourable efficacy outcome on Regimen C, the fully oral regimen, is non-inferior to that on Regimen B (with moxifloxacin or levofloxacin) at Week 76, using a 10% margin of non-inferiority

3.2 STREAM Stage 2 secondary objectives:

The secondary objectives of the Stage 2 comparison of the STREAM trial are:

1. To assess whether Regimen C is superior to Regimen B with regards to the proportion of participants with a favourable efficacy outcome at Week 76 and Week 132 (if non-inferiority is demonstrated at either time-point)

2. To assess whether Regimen C is non-inferior to Regimen B with regards to the proportion of participants with a favourable efficacy outcome at Week 132

3. To compare the efficacy of 40 weeks of bedaquiline in combination with the other drugs of Regimen C with Regimen B during treatment and follow-up

4. To compare the efficacy of 28 weeks of bedaquiline in combination with the other drugs of Regimen D with Regimen B during treatment and follow-up

5. To compare the safety, including the effect on mortality and tolerability, of 40 weeks of bedaquiline in combination with the other drugs of Regimen C with Regimen B during treatment and follow-up.

6. To compare the proportion of participants who experience grade 3 or greater adverse events during treatment or follow-up in Regimen C as compared to Regimen B

7. To estimate the difference between Regimen C and Regimen Bmox in the proportion of participants with a favourable efficacy outcome at Week 76 and Week 132

8. To estimate the difference between Regimen C and Regimen Blev in the proportion of participants with a favourable efficacy outcome at Week 76 and Week 132

9. To estimate the difference between Regimen C and Regimen A in the proportion of participants with a favourable efficacy outcome at Week 132

10. To estimate the difference between Regimen B and Regimen A in the proportion of participants with a favourable efficacy outcome at Week 132

11. To estimate the difference between Regimen D and Regimen B in the proportion of participants with a favourable efficacy outcome at Week 76 and Week 132

12. To estimate the difference between Regimen D and Regimen A in the proportion of participants with a favourable efficacy outcome at Week 132

13. To investigate the safety, including the effect on mortality and tolerability of 28 weeks of bedaquiline in combination with the other drugs of Regimen D compared to Regimen B during treatment and follow-up.

14. To compare the proportion of participants who experience grade 3 or greater adverse events during treatment or follow-up in Regimen B as compared to Regimen A

15. To compare the proportion of participants who experience grade 3 or greater adverse events during treatment or follow-up in Regimen C as compared to Regimen Bmox

16. To compare the proportion of participants who experience grade 3 or greater adverse events during treatment or follow-up in Regimen C as compared to Regimen Blev

17. To compare the proportion of participants who experience grade 3 or greater adverse events during treatment or follow-up in Regimen D as compared to Regimen B

18. To investigate the safety, including the effect on mortality and tolerability, of bedaquiline-containing regimens compared to Regimen B during treatment and follow-up.

19. To investigate the effect on mortality of bedaquiline-containing regimens compared to non-bedaquiline containing regimens.

20. To evaluate the pharmacokinetics of bedaquiline and M2 in all participants randomised to Regimen C or Regimen D at sites selected for the PK study and assess pharmacokinetic/pharmacodynamics relationships of bedaquiline for safety and efficacy

21. To evaluate the pharmacokinetics of bedaquiline and M2 in a subset of HIV co-infected participants on Regimen C or Regimen D receiving antiretroviral treatment

22. To evaluate the 4β-hydroxycholesterol/cholesterol ratio as a measure of cytochrome P450 3A (CYP3A) activity in Regimen C and Regimen D as compared to Regimen B.

23. To compare the economic costs incurred during treatment by patients (transport and food costs for attending DOTs and patient assessment visits, food supplements costs and income loss) and by the health system (inpatient stay, laboratory tests, medication, staff, consumables and serious adverse events costs) in Regimen B and C. To calculate economic costs associated with regimen D, and compare these with regimen B, for those sites where this is possible.

24. To compare the proportions of participants having undergone lung surgery (resection or pneumonectomy) by Week 76 and Week 132 in Regimen C and Regimen D as compared to Regimen B.

25. To compare the development of resistance to background drugs, especially resistance leading to the development of pre-XDR or XDR strains of TB in Regimen C and Regimen D as compared to Regimen B.

26. To investigate the development of increased MIC to bedaquiline in regimens C and D.

4 Detailed Methods

4.1 Randomisation

Participants were randomised in a ratio of 1:2:2:2 to the Long, Control, Oral and Six-month regimens; after randomisation to the Long regimen ceased in April 2018, the randomisation ratio to the three remaining

arms was equal. Randomisations were stratified by site and HIV status and CD4 count. Separate randomisation lists for each combination of strata were prepared by an independent statistician using permuted blocks of varying sizes. Participants were randomised using a web-based randomisation system; if web access was not available at the time of randomisation, a manual alternative using sealed envelopes was provided.

4.2 Interventions

Long Regimen: The Long regimen was the locally-used WHO 2011 long regimen.

Control regimen:

The Control consisted of moxifloxacin or levofloxacin (depending on protocol version), clofazimine, ethambutol and pyrazinamide given for 40 weeks, supplemented by kanamycin, isoniazid and prothionamide in the first 16 weeks. All drugs were administered orally except for kanamycin which was administered by intramuscular injection.

Product	Weight group		
	**Less than 33 kg**	**33 kg to 50 kg**	**More than 50 kg**
Moxifloxacin (protocols 6 & 7)<br>Levofloxacin (protocol 8 onwards)	400 mg<br>750 mg	600 mg<br>750 mg	800 mg<br>1000 mg
Clofazimine	50 mg	100 mg	100 mg
Ethambutol	800 mg	800 mg	1200 mg
Pyrazinamide	1000 mg	1500 mg	2000 mg
Isoniazid	300 mg	400 mg	600 mg
Prothionamide	250 mg	500 mg	750 mg
Kanamycin [1,2]	15 mg per kilogram body weight (maximum 1g)		

1. Protocol version 10 onwards, amikacin was to be used instead of kanamycin if the country's National Treatment Programme (NTP) had switched to amikacin, but this did not occur.

2. Given daily initially and then thrice-weekly from Week 12 onwards

All drugs were given daily (seven days a week), except for kanamycin; after the first 4 weeks of treatment was completed, in sites where it is not possible to administer kanamycin seven days a week to outpatients, the dose could be changed to six days a week. From week 12 onwards kanamycin frequency was reduced to three days a week.

The intensive phase should be extended from 16 to 20 or 24 weeks for participants whose smear has not converted by 16 or 20 weeks, respectively.

Oral regimen:

The Oral regimen was a modification of the Control regimen, and consisted of bedaquiline, clofazimine, ethambutol, levofloxacin, and pyrazinamide given for 40 weeks, supplemented by isoniazid and prothionamide in the first 16 weeks (intensive phase). All drugs were administered orally.

Product	Weight group		
	Less than 33 kg	33 kg to 50 kg	More than 50 kg
Bedaquiline	400 mg once daily for first 14 days/200 mg thrice weekly thereafter		
Levofloxacin	750 mg	750mg	1000 mg
Clofazimine	50 mg	100 mg	100 mg
Ethambutol	800 mg	800 mg	1200 mg
Pyrazinamide	1000 mg	1500 mg	2000 mg
Isoniazid	300 mg	400 mg	600 mg
Prothionamide	250 mg	500 mg	750 mg

All drugs were given daily (seven days a week), except for bedaquiline which was given daily for the first two weeks and then thrice-weekly from Week 2 onwards. The intensive phase should be extended from 16 to 20 or 24 weeks for participants whose smear has not converted by 16 or 20 weeks, respectively. If the intensive phase was extended beyond 16 weeks, then the overall treatment time was also extended except for bedaquiline, which was not to be given for longer than 40 weeks in total.

Six-month regimen

The Six-month regimen consisted of bedaquiline, clofazimine, levofloxacin, and pyrazinamide given for 28 weeks, supplemented by kanamycin and isoniazid in the first eight weeks (intensive phase). All drugs were administered orally except for kanamycin which was administered by intramuscular injection. All drugs were given daily (seven days a week) except for bedaquiline and isoniazid which were given daily for the first two weeks and then thrice-weekly from Week 2 onwards. After the first 4 weeks of treatment was completed, in sites where it was not possible to administer kanamycin seven days a week to outpatients the dose could be changed to six days a week from weeks 5-8. The intensive phase should be extended from 8 to 12 weeks and 12 to 16 weeks for participants with a smear positive of 2+ or more at 8 or 12 weeks, respectively. If the intensive phase was extended, kanamycin was to be given thrice-weekly from Week 8 onwards.

Product	Weight group				
	Less than 33 kg	33 kg to less than 40 kg	40 kg to 50 kg	More than 50 kg to 60 kg	More than 60 kg
Bedaquiline	400 mg once daily for first 14 days/200 mg thrice weekly thereafter				
Levofloxacin	750 mg	750 mg		1000 mg	
Clofazimine	50 mg	100 mg		100 mg	
Pyrazinamide	1000 mg	1500 mg		2000 mg	
Isoniazid	400 mg	500 mg	600 mg	800 mg	900 mg

	Daily for the first 14 days, thrice-weekly thereafter for the duration of the intensive phase
Kanamycin	15 mg per kilogram body weight (maximum 1g)

4.3 Participant full eligibility criteria

There were some minor modifications to the eligibility criteria during recruitment; full eligibility criteria from the final version of the protocol (version 11.0) are described here.

4.3.1 Inclusion criteria

A participant was eligible for randomisation into the study if he/she:

1.Was willing and able to give informed consent to participate in the trial treatment and follow-up (signed or witnessed consent if the patient is illiterate). If the patient was below the age of consent (according to local regulations), the parent/caregiver gave consent, and the patient was informed about the study and asked to give positive assent.
2. Was aged 15 years or older, unless that was not approved by the local ethics committee, in which case participants were required to be 18 years of over.
3. Had a positive AFB sputum smear result at screening (at least scanty), or a positive GeneXpert result (with a cycle threshold (Ct) value of 25 or lower) from a test performed at screening or from a test performed within the four weeks prior to screening
4. Had evidence of resistance to rifampicin either by line probe assay (Hain Genotype22), GeneXpert or culture-based drug susceptibility testing (DST), from a test performed at screening or from a test performed within the four weeks prior to screening
5. Was willing to have an HIV test and, if positive, was willing to be treated with ART in accordance with the national policies but excluding ART contraindicated for use with bedaquiline
6. Was willing to use effective contraception: pre-menopausal women or women whose last menstrual period was within the preceding year, who had not been sterilised must agree to use a barrier method or an intrauterine device unless their partner had had a vasectomy; men who had not had a vasectomy must agree to use condoms. Pre-menopausal women or women whose last menstrual period was within the preceding year, who had not been sterilised must agree to use two methods of contraception, for example a hormonal method and a barrier method
7. Resided in the area and was expected to remain for the duration of the study.
8. Had a chest X-ray that is compatible with a diagnosis of pulmonary TB (if such a chest X-ray taken within 4 weeks of randomisation is available, a repeat X-ray is not required)
9. Had normal K+, Mg2+ and corrected Ca2+ at screening.

4.3.2 Exclusion criteria

A participant was ineligible for randomisation into the study if he/she:

1. Was infected with a strain of M. tuberculosis resistant to second-line injectables by line probe assay (Hain Genotype22) from a test performed at screening or from a test performed within the four weeks prior to screening
2. Was infected with a strain of M. tuberculosis resistant to fluoroquinolones by line probe assay (Hain Genotype22) from a test performed at screening or from a test performed within the four weeks prior to screening
3. Had tuberculous meningitis or bone and joint tuberculosis
4. Was critically ill, and in the judgment of the investigator, unlikely to survive more than 4 months
5. Was known to be pregnant or breast-feeding
6. Was unable or unwilling to comply with the treatment, assessment, or follow-up schedule
7. Was unable to take oral medication
8. Had AST or ALT more than 3 times the upper limit of normal

9. Had any condition (social or medical) which in the opinion of the investigator would make study participation unsafe
10. In the investigator's opinion the patient was likely to be eligible for treatment with bedaquiline according to local guidelines due to a pre-existing medical condition, such as hearing loss or renal impairment
11. Was taking any medications contraindicated with the medicines in any trial regimen
12. Had a known allergy to any fluoroquinolone antibiotic or had previously experienced any serious adverse reaction when taking a quinolone or fluoroquinolone
13. Was currently taking part in another trial of a medicinal product
14. Had a QT or QTcF interval at screening or immediately prior to randomisation of more than or equal to 450 ms
15. Had experienced one or more of the following risk factors for QT prolongation:
  - Confirmed prolongation of the QT or QTcF more than or equal to 450 ms in the screening ECG (retesting to reassess eligibility will be allowed once using an unscheduled visit during the screening phase)
  - Pathological Q-waves (defined as Q-wave more than 40 ms or depth more than 0.4-0.5 mV)
  - Evidence of ventricular pre-excitation (e.g., Wolff Parkinson White syndrome)
  - Electrocardiographic evidence of complete or clinically significant incomplete left bundle branch block or right bundle branch block
  - Evidence of second- or third-degree heart block
  - Intraventricular conduction delay with QRS duration more than 120 ms
  - Bradycardia as defined by sinus rate less than 50 bpm
  - Personal or family history of Long QT Syndrome
  - Personal history of cardiac disease, symptomatic or asymptomatic arrhythmias, with the exception of sinus arrhythmia
  - Syncope (i.e. cardiac syncope not including syncope due to vasovagal or epileptic causes) Risk factors for Torsades de Pointes (e.g., heart failure, hypokalaemia, or hypomagnesemia)

16. Had received treatment for MDR-TB in the 12 weeks prior to screening, other than the maximum permitted treatment specified in Section 5.2.1 of the protocol
17. Had a history of cirrhosis and classified as Child's B or C at screening or a bilirubin more than 1.5 times upper limit of normal.
18. Had an estimated creatinine clearance (CrCl) less than 30 mL/min based on the Cockcroft-Gault equation
19. Was HIV positive and had a CD4 count less than 50 cells/mm3
20. Had pancreatic amylase elevation more than two times above the upper limit of normal
21. Had a history of alcohol and/or drug abuse
22. Had previous treatment with bedaquiline
23. Had taken rifampicin in the seven days prior to randomisation
24. There had been a delay of more than four weeks between the screening consent and randomisation
25. Was an employee or family member of the investigator or study site staff with direct involvement in the proposed study.

4.4 Study procedures

Consenting participants were assessed at screening, randomisation (Week 0), Week 1, Week 2, Week 3, Week 4, after which they were seen 4-weekly until Week 52, then 8-weekly until Week 84, and 12-weekly thereafter until end of trial follow-up.

A blood sample for hepatitis A (IgM), hepatitis B surface antigen (HBsAg), hepatitis B core antibody (HBcAb) and hepatitis C virus (HCV) antibody testing was collected at the randomisation visit.

Blood samples for complete blood count (CBC) and serum chemistry were measured at every scheduled evaluation (other than at weeks 1, 2, and 3) until the week 76 visit (as referred to as 'laboratory safety tests' below). After Week 76, blood tests were be undertaken if clinically indicated. All trial-specific samples were processed and analysed centrally or at appropriately qualified local laboratories.

ECG monitoring for Stage 2 involves 12-lead ECGs undertaken at baseline (pre and 4 hours post-dose at the randomisation visit), weekly for the first four weeks and at every visit until week 76. All participants whose QTcF at week 76 was higher than their baseline recording continued to have 12-lead ECG monitoring at every visit until the confirmed QTcF was either less than 10 ms above the baseline value or below 450 ms. ECGs were reviewed and reported by a centralised cardiology service.

Baseline chest radiographs were collected centrally and read by two independent clinicians, discordant assessments were read by a third clinician following published procedures.[1]

A central medical team with expertise in MDR-TB, HIV, clinical microbiology and electrocardiology was set up to advise investigators, when requested, in management of adverse events, guidance in management of participants needing retreatment and the promotion of consistent practice across the sites.

4.5 Bacteriological procedures

A minimum of two sputum specimens were collected at the screening and randomisation visit (with a third being an early morning sample if possible) for smear, culture and Hain Genotype MTBDRPlus line probe assay (LPA) . Two sputum specimens were collected at every subsequent visit for smear and culture, except at Week 1, Week 2, and Week 3. Early morning samples were preferred. Routine sputum collection ended when the last participant reached Week 96. Thereafter, sputum was collected only if clinically indicated. All specimens were tested for AFB smear and culture. If a participant was unable to produce sputum this was documented on the CRF.

The following bacteriological tests were performed at the site microbiology laboratory: smear, culture, diagnostic line probe assays or GeneXpert for rifampicin resistance and line probe assay for excluding resistance to second line drugs.

The selected methods and techniques for use by the sites were:

- Hot Ziehl-Neelsen (ZN) or Auramine O LED fluorescence methods for all study smears[2-4]
- FDA (Fluoresceine diacetate) vital staining for selected smears at trainedsites[5, 6]
- Culture on acidified Ogawa medium (Kudoh method without neutralisation centrifugation during decontamination and direct inoculation) for isolation of mycobacteria and subsequent identification of all positive study cultures using any locally available method (e.g. GeneXpert, Hain Genotype MTBDRplus, SD Bioline)[7-11]
- Hain Genotype MTBDRPlus line probe assay (LPA) from smear-positive sputum or GeneXpert System (Cepheid automated diagnostic test to identify rifampicin resistant Mycobacterium tuberculosis) for screening of participants with suspected RR-TB.[8-10] If one of these tests or other DST showed at least resistance to rifampicin, the Hain Genotype MTBDRsl LPA was performed to exclude fluoroquinolone and second-line injectable resistance.[12]

To increase the probability of having at least one good baseline isolate, the sites inoculated the remaining part of the randomisation and screening samples using their preferred culture method and medium (e.g. BD BACTEC™ MGIT™ 960 Mycobacteria Culture Systemafter neutralization and centrifugation).

All positive isolates, except those of week 4, were sent to the designated study reference laboratory, to confirm species identification and susceptibility status. This included diagnostic strains and recurrence strains, in case of failure or relapse besides isolated positive cultures in-between successive negatives. Strains from recurrences were tested for DST as well as fingerprinting, to confirm their identity and to

compare their resistance pattern with the originally isolated strain. The reference laboratory stored all study strains at -80°C and local laboratories stored at -20°C.

The techniques used at the reference laboratory were:

- SD Bioline Ag TB MPT64 Rapid test and sensitivity test to para-nitro benzoic acid (PNB) to exclude mixed infection with NTM.[11, 13]
- Slow phenotypic DST using the proportion method on Löwenstein-Jensen medium for first line drugs[14] [15, 16]
- Agar-based Middlebrook 7H11 medium for second line drugs,[15, 16]
- Bedaquiline MIC and clofazimine MIC on Middlebrook 7H11 agar medium were performed on all diagnostic samples (screening, randomisation and post-randomisation) ;
- discordances between the LPA results at the sites and the phenotypic DST in the reference laboratory were resolved using Target sequencing (Sanger) of the corresponding genes responsible for resistance.MIC Determination on Middlebrook 7H10 Agar for LFX and MFX against *M. tuberculosis* for paired baseline (at screening or enrolment) and recurrences (failures and relapses)[17].
- Fingerprinting; MIRU-VNTR analysis (mycobacterial interspersed repetitive units–variable number of tandem repeats).[18, 19]
- Resazurin Microtiter Assay (REMA) to determine the susceptibility/resistance of the strains to anti-tuberculous drugs (KM, CM, AM, and LZD) on paired isolates baseline (enrolment or screening) and failures or relapses.[20]

4.6 Assessment Schedule

Observation/Investigation	Screening	Randomisation	Treatment Phase			Post-Treatment Phase
			Intensive Phase		Continuation Phase	Follow-up
			Weeks 1 – 3	Weeks 4 onwards		
Written informed consent	X	X				
Demographics	X	X				
Medical History	X	X				
Alcohol Use Questionnaire		X		Week 16	Week 32	Week 52
Clinical Examination	X	X	X	X	X	X
Clinical assessment (including AEs and concomitant medication during treatment)	X	X	X	X	X	X
Height		X				
Weight	X	X	X	X	X	X
Visual acuity and colour tests		X		Week 12<br>(and if symptoms)	Week 28 & 40<br>(and if symptoms)	
Hearing test	X		Week 1<br>(If clinically indicated)	Week 4, 8 & 16	At the start of the continuation phase[11], Week 28 & 40	Weeks 52, 76 & 132
Haemoglobin		X				
HIV antibody test	X					Week 76[15]
CD4 (in HIV positive patients)	X		According to national guidelines, at end of BDQ dosing and at week 132			
Viral load (in HIV positive patients)	X			X[13]	X[13]	X[13, 15]
Hepatitis A, B and C testing		X				
Urinalysis (sample sent to) central lab		X	X	X	X	X[17]
Urine: HCG Pregnancy test	X	X	If clinically indicated and at end of study			
Chest X-ray[14]	X					

ECG (12-Lead)[3]	X	X	X	X	X	X
Additional Post-Dose ECG (12 Lead) for sites in PK study[4]			Week 2	Week 12	Weeks 24 & 40	
Sputum smear and culture[2]	X[1]	X[1]		X[1]	X[1]	X[1]
Sputum for drug resistance testing	X[6]					
Patient's costs (in selected sites)		X		X[12]	X[12]	X[12]
Blood sample for storage (if consents)[5]		X[5]			X[5]	
PK samples[7,8,9,18]		X	Week 2	Weeks 4 & 12	Weeks 24 & 40	Weeks 76, 120 & 132
Laboratory safety tests[10]	X	X		X	X	X[16]
TSH & thyroxine of free thyroxine	X					Weeks 40 & 76

X indicates assessments required at particular visits

[1] At screening and randomisation two samples will be collected, with an additional third early morning sample if possible. Two samples will be collected at each subsequent visit, ideally one early morning and one spot sample, or two spot samples if the patient does not provide an early morning sample. Refer to the STREAM Microbiology Manual for details of the tests to be undertaken.

[2] Screening, randomisation, and all positive isolates of MTB post-randomisation from week 8 onwards will be shipped to the reference laboratory for full drug susceptibility testing.

[3] An ECG will be conducted prior to randomisation, a further ECG will then be conducted 4 hours after administering treatment at the randomisation visit. A 12-lead ECG will then be collected at each visit until Week 76. In participants who at Week 76 have a QTcF increase from baseline, a 12-lead ECG will be collected at each visit until the QTcF returns to either less than a 10ms increase above the baseline value or less than 450 ms. Single ECGs will be collected; however for QTcF prolongatons of more than or equal to 500 ms, two further ECGs must be collected.

[4] For patients on arms C and D, enrolled at sites that have been pre-selected for the PK sub-study, an additional 12-lead ECG will also be conducted 4 hours after administering treatment at the week 2, 12, 24 and 40 visits

[5] A blood sample will be collected for storage at randomisation and week 16 , for patients consenting/assenting to sample storage.

[6] Sputum will be collected for drug sensitivity testing for resistance to rifampicin, fluoroquinolones and second-line injectables. If LPA results for fluoroquinolones and second-line injectables sensitivity are inconclusive, then these tests need to be repeated on a new sputum sample before randomisation.

[7] The PK samples will be collected pre-dose and post-dose (sample from Week 2 visit). Details of PK sampling are specified in section 8.2.1.

[8] Samples for analysis of the plasma concentration of nevirapine (NVP) and lopinavir (LPV)/ritonavir(RTV) must be taken before intake of ARV and study drug. An additional pre-dose sample will be collected if the antiretroviral

treatment regimen of a patient is changed, followed by sampling at time points indicated in the Assessment Schedule.

[9] Sample for analysis of the plasma concentration of nevirapine (NVP) and lopinavir (LPV)/ritonavir (RTV) and 4 β OH-cholesterol.

[10] See Section 8.2 for blood test details.

[11] Hearing test will be conducted at the first visit of the continuation phase.

[12] Patient costs collected every 12 weeks from after randomisation in selected sites.

[13] Viral load collected at Week 12, Week 24, Week 40, and Week 76.

[14] A chest X-ray is required at randomisation that is compatible with a diagnosis of pulmonary TB, however if a good quality X-ray is available that was taken in the 4 weeks prior to randomisation it does not need to be repeated

[15] HIV test at week 76 (for patients who were found to be HIV negative at screening). For patients found to be HIV positive at this visit a week 76 viral load measurement should also be taken.

[16] Laboratory safety tests should be undertaken at each visit to Week 76. After Week 76 only if clinically indicated.

[17] Urinalysis to central lab should be undertaken at each visit to Week 76. After Week 76 only if clinically indicated.

[18] Samples will not be taken after the projected Week 96 visit of the last patient randomised i.e. approximately end of November 2021.

4.7 Analysis definitions

4.7.1 Analysis Populations

4.7.1.1 Intention-to-treat (ITT)

All randomised patients will be included in the ITT analysis population.

4.7.1.2 Modified intention-to-treat (mITT)

The mITT population is defined as all randomised participants that have a positive culture for *M. tuberculosis* at screening or randomisation, with the exception of participants with isolates taken before randomisation that are subsequently found to be susceptible to rifampicin, and patients with isolates taken before randomisation that are subsequently found to be resistant to both fluoroquinolones and second-line injectables (i.e. XDR-TB) on phenotypic DST. Results from the central reference laboratory take priority over any results from local laboratories where available. Genotypic DST from the central reference laboratory were used when phenotypic DST was unavailable. Rifampicin susceptible phenotypic DST results were confirmed by rpoB sequencing. Participants randomised in error i.e. late screening failures, are excluded from the mITT population.

3.7.1.3 Per protocol (PP)

The PP population is the same as the mITT population with the exclusion of participants not completing a protocol-adherent course of treatment, other than for treatment failure, change of treatment for an adverse event or death. Treatment failure was defined as failure to attain and maintain culture negativity until the end of allocated treatment.

4.7.1.4 Safety population
All randomised participants that have taken at least one dose of treatment are included in the safety analysis population.

4.7.2 Protocol adherent treatment

For the Control regimen and Oral regimen, with or without an extension of the intensive phase, a participant was defined as having completed a protocol-adherent course of treatment if they had taken 80% of doses within 120% of weeks. For example, in participants with no treatment extensions:

- 90 doses (80% of 16 weeks) within 134 days (120% of 16 weeks) in the intensive phase, and
- 224 doses (80% of 40 weeks) within 336 days (120% of 40 weeks) over the whole treatment period (i.e. the combined intensive and continuation phases)

or in participants with a four week extension:

- 112 doses (80% of 20 weeks) within 168 days (120% of 20 weeks) in the intensive phase, and
- 246 doses (80% of 44 weeks) within 370 days (120% of 44 weeks) over the whole treatment period (i.e. the combined intensive and continuation phases).

For the Six-month regimen, with or without an extension of the intensive phase, a participant was defined as having completed a protocol-adherent course of treatment if they had taken 80% of doses within 120% of weeks. For example, in participants with no treatment extensions:

- 45 doses (80% of 8 weeks) within 67 days (120% of 8 weeks) in the intensive phase, and
- 157 doses (80% of 28 weeks) within 235 days (120% of 28 weeks) over the whole treatment period (i.e. the combined intensive and continuation phases)

or in participants with a four week extension:

- 67 doses (80% of 12 weeks) within 101 days (120% of 12 weeks) in the intensive phase, and
- 179 doses (80% of 32 weeks) within 269 days (120% of 32 weeks) over the whole treatment period (i.e. the combined intensive and continuation phases).

4.7.3 Primary efficacy outcome

4.7.3.1 Favourable

A participant’s outcome was classified as favourable if their last two microbiological culture results were negative unless they had previously been classified as unfavourable. These two cultures must be taken on separate visits (on different days); the latest of which being within the Week 76 window.

Participants that did not have a culture result within the Week 76 window because they were unable to produce sputum or their sample was contaminated, were classified as favourable provided their last two

cultures before the Week 76 window were negative and they had not previously been classified as unfavourable.

The end dates of the Week 76 window were extended for any Stage 2 patients whose Week 76 appointment was scheduled to occur during the COVID-19 pandemic and did not occur due to restrictions on movement, unacceptable risk of exposure to COVID-19 in connection with the scheduled visit, or any other reason related to the pandemic. For those patients, the Week 76 sputum samples had to be taken in a window beginning six weeks prior to the scheduled visit date and ending within the Week 84 visit window i.e. within 14 weeks of the scheduled Week 76 visit date.

Participants with no sputum sample available at Week 76 due to Covid-19 restrictions who are not otherwise classified as unfavourable were considered unfavourable. Sensitivity analyses reclassified these participants as i) non-assessable and excluded from the primary efficacy analysis, and ii) favourable if they met the definition of favourable above, with the latest of the 2 negative culture results being within the Week 68 window, and unfavourable otherwise.

Only 2 participants had an extended Week 76 window, and 1 participant missed their Week 76 visit due to the COVID-19 pandemic.

4.7.3.2 Unfavourable

A participant's outcome was classified as unfavourable if:

- They were discontinued from their allocated study treatment and subsequently restarted on a different MDR-TB regimen.
- Treatment was extended beyond the scheduled end of treatment for any reason other than making up days when no treatment was given (missed treatment) for a maximum of eight weeks. A maximum of 14 days of extra treatment (irrespective of reason) was acceptable before it was classified as treatment extension.
- They were restarted on any MDR-TB treatment after the scheduled end of treatment, but before 76 weeks after randomisation.
- They changed their allocated study treatment for any reason other than the replacement of a single drug.
- Bedaquiline was started where the allocated regimen did not originally contain that drug (Long regimen or Control regimen).
- A second line injectable agent was started in the Oral regimen.
- A drug from the class of nitroimidazoles (delamanid or pretomanid) or linezolid was started.
- They died at any point during treatment or follow-up.
- At least one of their last two culture results, from specimens taken on separate occasions, was positive.
- They did not have a culture result within the Week 76 window.

Starting a single drug other than bedaquiline (in the Control regimen) or from the class of nitroimidazoles (delamanid or pretomanid) or linezolid (in any regimen) was not considered to be a substantial change to the regimen and therefore did not result in an unfavourable outcome, providing none of the other criteria above were met.

Initiating linezolid was added as an unfavourable outcome in version 11.0 of the protocol, which was approved in December 2020. After publication of the revised WHO guidelines in 2019,[21] in which it was

recommended that bedaquiline, linezolid, and either moxifloxacin or levofloxacin should be used in MDR treatment regimens and kanamycin should be avoided, it became more likely that a treatment change for those initially allocated to the Oral regimen would be to add linezolid, rather than kanamycin or another injectable agent should bedaquiline have to be stopped.

An extension of the intensive phase of treatment in any study arm was not considered to constitute an unfavourable outcome, as long as the extension was in accordance with the protocol-specified treatment extension for a positive smear at the end of the intensive phase. Similarly, the discontinuation of drugs that were not replaced did not constitute an unfavourable outcome.

These definitions of favourable and unfavourable applied to all three treatment regimens and for analyses of both the mITT and PP analysis populations.

4.7.5 Analysis of culture results

A culture result will be called positive for M. tuberculosis if the culture tests positive for the presence of microorganisms, at least one colony, and the microorganisms present are then identified as being M. tuberculosis. Identification was based on tests performed at the central laboratory (ITM). If an identification test was not carried out for a particular culture, then for analysis purposes a culture was still considered positive for M. tuberculosis if the culture tested positive for the presence of microorganisms and if that culture result was obtained fourteen days or more since the start date of sputum processing and incubation of the inoculated Ogawa or Löwenstein-Jensen (LJ) media. If the culture result was obtained less than fourteen days since the start date of sputum processing and incubation, the culture result was not considered as positive for M. tuberculosis (if the identification test was not carried out), and the culture result was considered missing in the analysis.

Culture results obtained using acidified Ogawa (Kudoh medium) are used in analysis if available; results from LJ media are used if the Ogawa result was missing. The only exception will be pre-randomisation when MGIT culture results will also be included in determining analysis populations.

Any culture result that is missing because the patient is no longer able to produce sputum is treated as a negative result, providing their last 2 available culture results (from sputum samples taken at separate visits) were negative.

5 Supplementary Tables and Figures

Table S1: Summary of participants screened and randomised by centre

Country	Site	Randomised	Screened
**Ethiopia**	Addis Ababa, AHRI	25	55
	Addis Ababa, St. Peters	42	68
**India**	Delhi	33	88
	Chennai	49	130
	Ahmedabad	66	223
**Mongolia**	Ulaanbatar	130	231
**Georgia**	Tbilisi	32	50
**Moldova**	Chisinau	63	130
**South Africa**	Pietermaritzburg	20	53
	Johannesburg	20	111
	Port Elizabeth	3	14
	Durban	49	153
**Uganda**	Kampala	56	130
	Total	588	1436

*85% of randomised participants started treatment the same day as randomisation, 15% started treatment one day after randomisation, 1 participant started 2 days after randomisation

Table S2: Summary of randomisation allocation by country, mITT population

Regimen	Ethiopia	India	Mongolia	Moldova	Georgia	South Africa	Uganda	Total
**Total**	61	138	125	60	32	73	54	543
**Long**	4 (7%)	3 (2%)	10 (8%)	4 (7%)	0	5 (7%)	0	26 (5%)
**Control MFX**	18 (30%)	42 (30%)	23 (18%)	8 (13%)	7 (22%)	22 (30%)	7 (13%)	127 (23%)
**Control LFX**	2 (3%)	0	22 (18%)	16 (27%)	6 (19%)	0	14 (26%)	60 (11%)
**Oral**	19 (31%)	46 (33%)	46 (37%)	24 (40%)	12 (38%)	25 (34%)	24 (44%)	196 (36%)
**Six-month**	18 (30%)	47 (34%)	24 (19%)	8 (13%)	7 (22%)	21 (29%)	9 (17%)	134 (25%)

Control MFX = participants randomised to the Control regimen including moxifloxacin, Control LFX = participants randomised to the Control regimen including levofloxacin

Table S3: Summary of Analysis Populations by treatment arm

N (% of total randomised)	Control	Oral	Control	Six-month
**Total Randomised**	202	211	140	143
**ITT population**	202 (100%)	211 (100%)	140 (100%)	143 (100%)
**Safety population**	202 (100%)	211 (100%)	140 (100%)	143 (100%)
**mITT population**	187 (93%)	196 (93%)	127 (91%)	134 (94%)
**mITT exclusion reasons**				
**RIF-susceptible**	5 (2%)	7 (3%)	5 (4%)	3 (2%)
**XDR**	1 (<0.5%)	2 (1%)	0	1 (1%)
**Negative baseline results at ITM**	0	0	0	1 (1%)
**No positive culture at baseline**	4 (2%)	6 (3%)	4 (3%)	2 (1%)
**Sample no longer available**	3 (1%)	0	3 (2%)	2 (1%)
**Randomised in error: ECG QTcF > 450ms**	1 (<0.5%)	0	0	0
**Randomised in error: Pre-existing hearing loss**	1 (<0.5%)	0	1 (<0.5%)	0
**PP population**	166 (82%)	177 (84%)	110 (79%)	122 (85%)
**PP exclusion reasons**				
**< 80% expected doses (Intensive Phase)**	6 (3%)	8 (4%)	5 (4%)	0
**< 80% expected doses (Overall)**	1 (<0.5%)	3 (1%)	1 (1%)	1 (1%)
**> 120% expected duration (Intensive Phase)**	4 (2%)	5 (2%)	3 (2%)	7 (5%)
**> 120% expected duration (Overall)**	3 (1%)	3 (1%)	1 (1%)	2 (1%)
**Started non-protocol treatment (Intensive Phase)**	2 (1%)	0	2 (1%)	1 (1%)
**Started non-protocol treatment (Overall)**	5 (2%)	0	5 (4%)	1 (1%)

Six-month participants are compared to the subset of Control participants who were randomised concurrently.

Table S4: Additional baseline characteristics of the mITT population

Baseline characteristic		Control	Oral	Control	Six-month
**Total in mITT population**		**187**	**196**	**127**	**134**
**Heart Rate (bpm)**	<60	7 (4%)	8 (4%)	4 (3%)	6 (4%)
	60-99	142 (76%)	136 (69%)	93 (73%)	87 (65%)
	≥100	38 (20%)	52 (27%)	30 (24%)	41 (31%)
**QT (ms)**	<400	163 (87%)	175 (89%)	115 (91%)	121 (90%)
	400-449	24 (13%)	21 (11%)	12 (9%)	13 (10%)
**QTcF (ms) (Fredericia's)**	<400	90 (48%)	107 (55%)	71 (56%)	89 (66%)
	400-449	96 (51%)	89 (45%)	55 (43%)	45 (34%)
	450-499	1 (1%)	0	1 (1%)	0
**Smear[1]**	No AFB Seen	9 (5%)	2 (1%)	4 (3%)	3 (2%)
	Rare AFB (1-9/100 fields)	16 (9%)	15 (8%)	12 (9%)	9 (7%)
	1+	37 (20%)	47 (24%)	24 (19%)	30 (22%)
	2+	43 (23%)	47 (24%)	26 (20%)	37 (28%)
	3+	82 (44%)	85 (43%)	61 (48%)	55 (41%)
**Culture[1]**	Positive	187 (100%)	196 (100%)	127 (100%)	134 (100%)
**Culture (colony count)[1]**	MGIT positive	11 (6%)	14 (7%)	11 (9%)	13 (10%)
	1-100 colonies	41 (22%)	46 (23%)	28 (22%)	31 (23%)
	2+	71 (38%)	64 (33%)	49 (39%)	43 (32%)
	3+	64 (34%)	72 (37%)	39 (31%)	47 (35%)
**ARV Regimen[2]**	No ART	1 (4%)	0	1 (5%)	0
	NVP	4 (16%)	13 (48%)	4 (19%)	10 (48%)
	LPVr	14 (56%)	10 (37%)	12 (57%)	11 (52%)
	DTG/RAL	3 (12%)	4 (15%)	1 (5%)	0
	Non permissible ART	3 (12%)	0	3 (14%)	0

Six-month participants are compared to the subset of Control participants who were randomised concurrently.
[1] Smear and culture results are based on local microbiology
[2] ARV regimen at randomisation or ARV regimen started within first 8 weeks of follow-up if off ART at randomisation.

Table S5: Retention at week 76

	Safety population	mITT population				
	Total	Control	Oral	Control	Six-month	Total
Total Expected	588	187	196	127	134	543
Seen here, sputum collected	542 (92%)	171 (91%)	177 (90%)[1]	115 (91%)	128 (96%)	502 (92%)
Seen here, sputum not collected	3 (1%)	1 (1%)	1 (1%)	1 (1%)	1 (1%)	3 (1%)
Not seen at this timepoint	11 (2%)	3 (2%)	4 (2%)	3 (2%)	2 (1%)	9 (2%)
Died	13 (2%)[1]	5 (3%)	5 (3%)[1]	2 (2%)	2 (1%)	12 (2%)[1]
Discontinued early	19 (3%)	7 (4%)	9 (5%)	6 (5%)	1 (1%)	17 (3%)

Six-month participants are compared to the subset of Control participants who were randomised concurrently.
[1] 1 participant attended week 76 sputum visit and later died within the week 76 window – reported in table as "Seen here, sputum collected"

Table S6: Adherence by treatment arm in mITT population

	Control	Oral	Control	Six-month
**0 – 74%**	1 (1%)	2 (1%)	1 (1%)	1 (1%)
**75 – 89%**	14 (7%)	5 (3%)	12 (9%)	2 (1%)
**90 – 94%**	7 (4%)	10 (5%)	3 (2%)	1 (1%)
**95 – 100%**	165 (88%)	178 (91%)	111 (87%)	130 (97%)
**Missing[1]**	0	1 (1%)	0	0
**Total**	187 (100%)	196 (100%)	127 (100%)	134 (100%)
**Median (IQR)**	100.0 (99.6, 100.0)	100.0 (99.6, 100.0)	100.0 (99.6, 100.0)	100.0 (100.0, 100.0)

Adherence was defined as the number of prescribed doses minus the number of doses self-reported as not taken on diary cards, divided by the number of prescribed doses.
Six-month participants are compared to the subset of Control participants who were randomised concurrently.
[1] Participant switched to national treatment program same day they started allocated regimen

Table S7: Primary outcome, per-protocol population

	Control	Oral	Control	Six-month
**Total randomised**	202	211	140	143
**Total in PP population**	166	177	110	122
**Total favourable**	126 (75.9%)	155 (87.6%)	82 (74.5%)	114 (93.4%)
**Total unfavourable**	40 (24.1%)	22 (12.4%)	28 (25.5%)	8 (6.6%)
**Determined on the basis of bacteriologic findings:**	**18 (45%)**	**7 (32%)**	**14 (50%)**	**3 (38%)**
**Died within 3 weeks of randomisation (culture positive)**	1	2	1	0
**Bacteriologic reversion during treatment period**	10	3	7	1
**Restarted treatment for bacteriologic recurrence**	1	1	1	1
**Changed treatment due to persistent positive culture during treatment period**	5	0	4	1
**Changed treatment due to tuberculosis empyema**	0	1	0	0
**Positive culture at week 76**	1	0	1	0
**Determined on the basis of non-bacteriologic findings:**	**22 (55%)**	**15 (68%)**	**14 (50%)**	**5 (62%)**
**Died (culture negative)**	1	3	0	2
**Lost to follow up before 76 weeks (culture negative)**	2	4	1	2
**Treatment changed after adverse event**	19	6	13	1
**Started ≥2 drugs**	1	0	1	0
**Started BDQ**	5	0	4	0
**Started KAN**	0	6	0	0
**Started LZD**	13	0	8	1
**Treatment extended after adverse event**	0	1	0	0
**Treatment extended due to investigator decision**	0	1	0	0
**Treatment changed for other reasons**	0	0	0	0

Table presents unfavourable outcomes that lead to the primary endpoint i.e. the first unfavourable event experienced by each participant who was classified as unfavourable. Six-month participants are compared to the subset of Control participants who were randomised concurrently.

Table S8: Acquired resistance on allocated treatment

Drug	Control	Oral	Control	Six-month
**Total unfavourable for bacteriological reason**	20	8	16	3
**No DST Results[1]**	1	1	1	0
**DST Results**	19	7	15	3
**Acquired resistance by drug**				
Bedaquiline + clofazimine	0	1	0	0
Clofazimine	1	1	1	0
Fluoroquinolones	1	1	1	3
Fluoroquinolones + clofazimine	0	1	0	0
Kanamycin	2	0	0	0
Pyrazinamide	1	0	1	0
**Any resistance**	5	4	3	3
**% of total in mITT**	5 (2.7%) /187	4 (2.0%) /196	3 (2.4%) /127	3 (2.2%) /134

A participant was determined to have acquired resistance to a particular drug if they were sensitive at baseline and had a non-isolated resistant DST result on a sample after baseline before starting salvage therapy. Phenotypic DST was used; if unavailable genotypic DST was used. Six-month participants are compared to the subset of Control participants who were randomised concurrently. DST = Drug susceptibility testing.

[1] 2 participants with no available DST died within 3 weeks of randomisation

Table S9 Difference in proportion with a favourable outcome: Sensitivity analyses for the primary outcome

Sensitivity Analysis	Difference (95% CI) : Control – Oral	P value for non-inferiority
**Statified by randomisation protocol only[1]**	-11.4% (-19.7%, -3.1%)	<0.0001
**Stratified by randomisation protocol and HIV status, adjusted for baseline smear, culture, age, isoniazid resistance, extend of opacity, and number of cavities[2]**	-10.3% (-18.8%, -1.7%)	0.019
**Ignoring substitutions of levofloxacin for moxifloxicin and vice versa within the definition of unfavourable outcome.**	-11.0% (-19.0%, -2.9%)	<0.0001
**Ignoring starting linezolid alone as a criterion for an unfavourable outcome**	-5.1% (-12.8%, 2.7%)	<0.0001
**Reclassifying any participants with missing Week 76 culture results because of COVID-19 as "non-assessable" rather than unfavourable.**	-11.1% (-19.1%, -3.0%)	<0.0001
**Reclassifying any participants with missing Week 76 culture results because of COVID-19 as favourable if they meet the definition of favourable by Week 68, unfavourable otherwise**	-11.0% (-19.0%, -2.9%)	<0.0001
**Requiring a 25 day delay between the last 2 negative culture results in participants classified as favourable.**	-11.0% (-19.0%, -2.9%)	<0.0001
**ITT population[3]**	-12.6% (-20.4%, -4.9%)	<0.0001
**Safety population[3]**	-12.6% (-20.4%, -4.9%)	<0.0001

[1] All other sensitivity analyses stratified by randomisation protocol and HIV status

[2] Adjusted difference in proportions estimated using logistic regression

[3] All other sensitivity analyses are for the mITT population

Figure S1 Difference in proportion with a favourable outcome: Subgroup analyses of the primary outcome

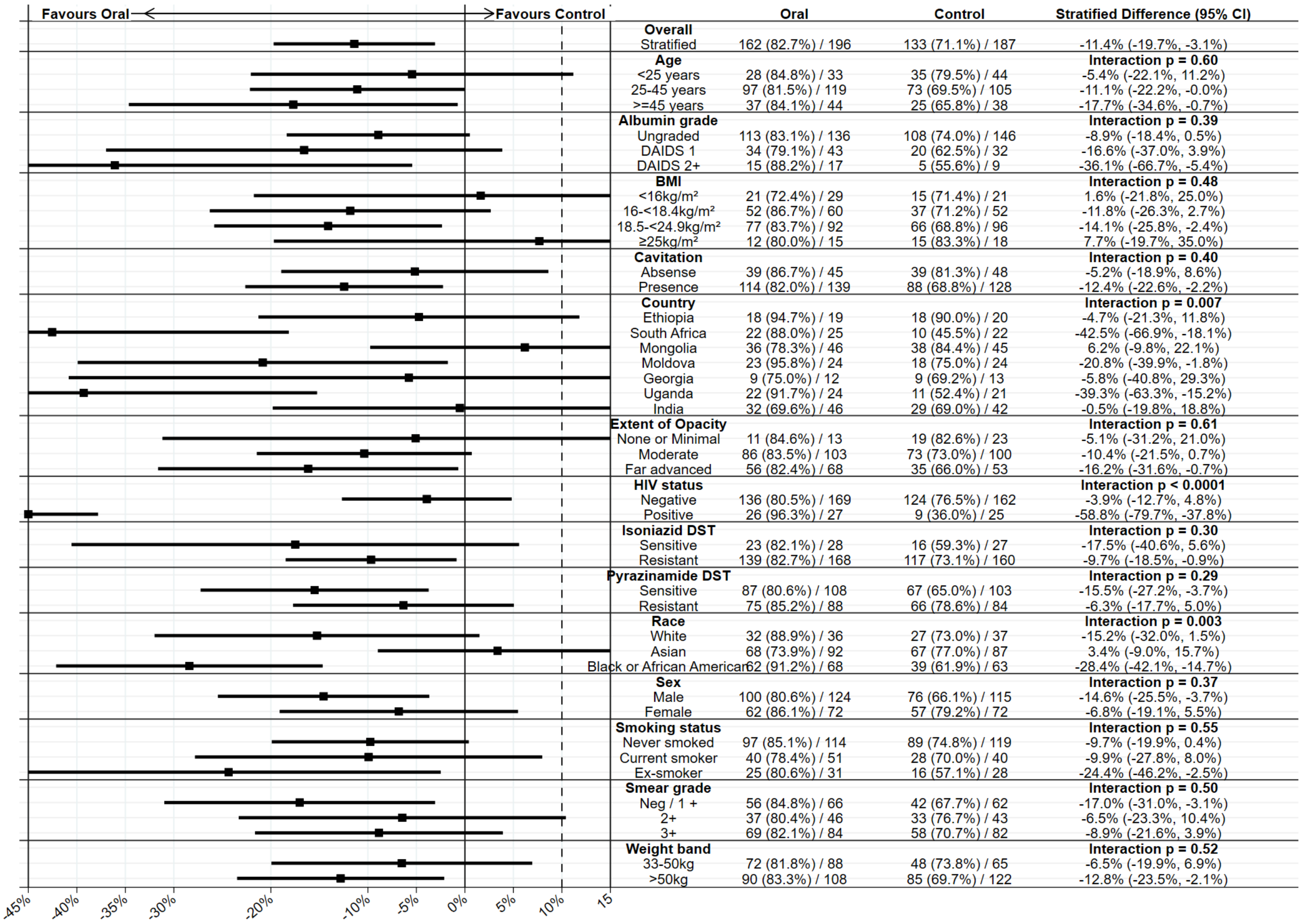

	Oral	Control	Stratified Difference (95% CI)
**Overall**			
Stratified	162 (82.7%) / 196	133 (71.1%) / 187	-11.4% (-19.7%, -3.1%)
**Age**			**Interaction p = 0.60**
<25 years	28 (84.8%) / 33	35 (79.5%) / 44	-5.4% (-22.1%, 11.2%)
25-45 years	97 (81.5%) / 119	73 (69.5%) / 105	-11.1% (-22.2%, -0.0%)
>=45 years	37 (84.1%) / 44	25 (65.8%) / 38	-17.7% (-34.6%, -0.7%)
**Albumin grade**			**Interaction p = 0.39**
Ungraded	113 (83.1%) / 136	108 (74.0%) / 146	-8.9% (-18.4%, 0.5%)
DAIDS 1	34 (79.1%) / 43	20 (62.5%) / 32	-16.6% (-37.0%, 3.9%)
DAIDS 2+	15 (88.2%) / 17	5 (55.6%) / 9	-36.1% (-66.7%, -5.4%)
**BMI**			**Interaction p = 0.48**
<16kg/m²	21 (72.4%) / 29	15 (71.4%) / 21	1.6% (-21.8%, 25.0%)
16-<18.4kg/m²	52 (86.7%) / 60	37 (71.2%) / 52	-11.8% (-26.3%, 2.7%)
18.5-<24.9kg/m²	77 (83.7%) / 92	66 (68.8%) / 96	-14.1% (-25.8%, -2.4%)
≥25kg/m²	12 (80.0%) / 15	15 (83.3%) / 18	7.7% (-19.7%, 35.0%)
**Cavitation**			**Interaction p = 0.40**
Absense	39 (86.7%) / 45	39 (81.3%) / 48	-5.2% (-18.9%, 8.6%)
Presence	114 (82.0%) / 139	88 (68.8%) / 128	-12.4% (-22.6%, -2.2%)
**Country**			**Interaction p = 0.007**
Ethiopia	18 (94.7%) / 19	18 (90.0%) / 20	-4.7% (-21.3%, 11.8%)
South Africa	22 (88.0%) / 25	10 (45.5%) / 22	-42.5% (-66.9%, -18.1%)
Mongolia	36 (78.3%) / 46	38 (84.4%) / 45	6.2% (-9.8%, 22.1%)
Moldova	23 (95.8%) / 24	18 (75.0%) / 24	-20.8% (-39.9%, -1.8%)
Georgia	9 (75.0%) / 12	9 (69.2%) / 13	-5.8% (-40.8%, 29.3%)
Uganda	22 (91.7%) / 24	11 (52.4%) / 21	-39.3% (-63.3%, -15.2%)
India	32 (69.6%) / 46	29 (69.0%) / 42	-0.5% (-19.8%, 18.8%)
**Extent of Opacity**			**Interaction p = 0.61**
None or Minimal	11 (84.6%) / 13	19 (82.6%) / 23	-5.1% (-31.2%, 21.0%)
Moderate	86 (83.5%) / 103	73 (73.0%) / 100	-10.4% (-21.5%, 0.7%)
Far advanced	56 (82.4%) / 68	35 (66.0%) / 53	-16.2% (-31.6%, -0.7%)
**HIV status**			**Interaction p < 0.0001**
Negative	136 (80.5%) / 169	124 (76.5%) / 162	-3.9% (-12.7%, 4.8%)
Positive	26 (96.3%) / 27	9 (36.0%) / 25	-58.8% (-79.7%, -37.8%)
**Isoniazid DST**			**Interaction p = 0.30**
Sensitive	23 (82.1%) / 28	16 (59.3%) / 27	-17.5% (-40.6%, 5.6%)
Resistant	139 (82.7%) / 168	117 (73.1%) / 160	-9.7% (-18.5%, -0.9%)
**Pyrazinamide DST**			**Interaction p = 0.29**
Sensitive	87 (80.6%) / 108	67 (65.0%) / 103	-15.5% (-27.2%, -3.7%)
Resistant	75 (85.2%) / 88	66 (78.6%) / 84	-6.3% (-17.7%, 5.0%)
**Race**			**Interaction p = 0.003**
White	32 (88.9%) / 36	27 (73.0%) / 37	-15.2% (-32.0%, 1.5%)
Asian	68 (73.9%) / 92	67 (77.0%) / 87	3.4% (-9.0%, 15.7%)
Black or African American	62 (91.2%) / 68	39 (61.9%) / 63	-28.4% (-42.1%, -14.7%)
**Sex**			**Interaction p = 0.37**
Male	100 (80.6%) / 124	76 (66.1%) / 115	-14.6% (-25.5%, -3.7%)
Female	62 (86.1%) / 72	57 (79.2%) / 72	-6.8% (-19.1%, 5.5%)
**Smoking status**			**Interaction p = 0.55**
Never smoked	97 (85.1%) / 114	89 (74.8%) / 119	-9.7% (-19.9%, 0.4%)
Current smoker	40 (78.4%) / 51	28 (70.0%) / 40	-9.9% (-27.8%, 8.0%)
Ex-smoker	25 (80.6%) / 31	16 (57.1%) / 28	-24.4% (-46.2%, -2.5%)
**Smear grade**			**Interaction p = 0.50**
Neg / 1 +	56 (84.8%) / 66	42 (67.7%) / 62	-17.0% (-31.0%, -3.1%)
2+	37 (80.4%) / 46	33 (76.7%) / 43	-6.5% (-23.3%, 10.4%)
3+	69 (82.1%) / 84	58 (70.7%) / 82	-8.9% (-21.6%, 3.9%)
**Weight band**			**Interaction p = 0.52**
33-50kg	72 (81.8%) / 88	48 (73.8%) / 65	-6.5% (-19.9%, 6.9%)
>50kg	90 (83.3%) / 108	85 (69.7%) / 122	-12.8% (-23.5%, -2.1%)

First two columns show number and percentage of favourable / assessable participants in MITT analysis population

All subgroup analyses are stratified by randomisation protocol, except for analyses by country which is not stratified due to differences in protocol implementation at country level. Differences by race are likely driven by country differences.

In the Failure or Recurrence (FoR) analysis, favourable outcome for each participant was re-classified according to the likelihood that it was a *Failure or Recurrence (FoR) event* on a five-point Likert scale: Definite, Probable, Possible, Unlikely, and Highly Unlikely.[22] An event was considered *Highly Unlikely* to be a FoR event only if there was evidence of durable cure; i.e. the primary outcome classification of favourable which required completion of follow-up with negative cultures. A *Definite* FoR event required clear bacteriological evidence of failure or recurrence (excluding a proven reinfection with exogenous strain of *M. tuberculosis)*, a *Probable* FoR event required some evidence for failure or recurrence (clinical, bacteriological, or radiological) in the absence of clear bacteriology. The remaining participants were classified as possible or unlikely based on their data. The FoR classification was reviewed by an independent clinician, blinded to treatment. In the time to FoR analyses, the main groups of interest are those classified as having a Definite or Probable FoR event, with censoring of Possible, Unlikely and Highly Unlikely events at the time of the censoring event which met criteria for Unfavourable in the primary analysis.

Table S10 Sensitivity analyses to account for informative censoring in time to FoR event

	Hazard ratio with 95% CI Control vs Oral	P-value
**Unadjusted, assuming independent censoring**	4.49 (1.51, 13.38)	0.0070
**Unadjusted, using IPCW**	5.14 (1.68, 15.66)	0.0042
**Adjusted for baseline covariates, assuming independent censoring**	5.12 (1.68, 15.70)	0.0041
**Adjusted, using IPCW with time varying covariates**	5.52 (1.84, 16.59)	0.0024
**Adjusted, using IPCW with no time varying covariates**	5.51 (1.84, 16.50)	0.0023

IPCW = Inverse probability censoring weighting. These analyses investigate the assumption that the likelihood for an FoR event at the time of censoring is the same as for those in whom no censoring occurred using IPCW. There is no substantial difference between the hazard ratios for each sensitivity analysis, therefore we conclude that censoring is independent of outcome.

Table S11 Predictors of probability of censoring used in sensitivity analyses of time to FoR event

Covariate	Level	Control	Oral
Time varying: cumulative number of grade 3-5 AEs	0	*Reference*	*Reference*
	1	1.19 (0.44, 3.22)	1.07 (0.28, 4.07)
	2	2.4 (0.82, 7.04)	5.31 (1.5, 18.83)
	3 or more	3.81 (1.06, 13.90)	14.9 (4.78, 46.42)
Time varying: most recent culture was positive		0.77 (0.64, 0.93)	0.78 (0.62, 0.98)
Baseline smear grading	Negative, Scanty, 1+	*Reference*	*Reference*
	2+	0.87 (0.37, 2.05)	1.06 (0.39, 2.93)
	3+	0.58 (0.28, 1.23)	1.22 (0.51, 2.91)
BMI at baseline, per 1 kg/m^2		0.99 (0.91, 1.08)	0.98 (0.88, 1.09)
Age at baseline, per 1 year		1.03 (1, 1.06)	1.01 (0.98, 1.05)
Continent	Asia	*Reference*	*Reference*
	Africa	3.6 (1.67, 7.75)	0.20 (0.07, 0.62)
	Europe	1.08 (0.42, 2.81)	0.3 (0.002, 0.33)

*Odds ratios adjusted for cubic spline (3 knots) of time-varying baseline hazard, HIV CD4 count, and protocol Investigating predictors of probability of censoring (Possible, Unlikely, or Highly Unlikely FoR events) within time interval from logistic regression weight determining model.

Time to sputum smear conversion was defined as the time from randomisation to the first of two consecutive negative sputum results, collected on separate days. All patients in the mITT analysis population were included in this analysis, except those with no positive smear result at screening and randomisation. Patients that never achieved smear conversion were censored at the date of collection of sputum that yielded their last smear result.

Table S12: Time to smear conversion: median survival time – Control vs Oral

	Median survival time (IQR), weeks
**Control**	7.14 (4.14, 12.14)
**Oral**	4.43 (4.14, 12.14)

Figure S2: Time to smear conversion: Kaplan-Meier survival estimates and hazard ratio – Control vs Oral

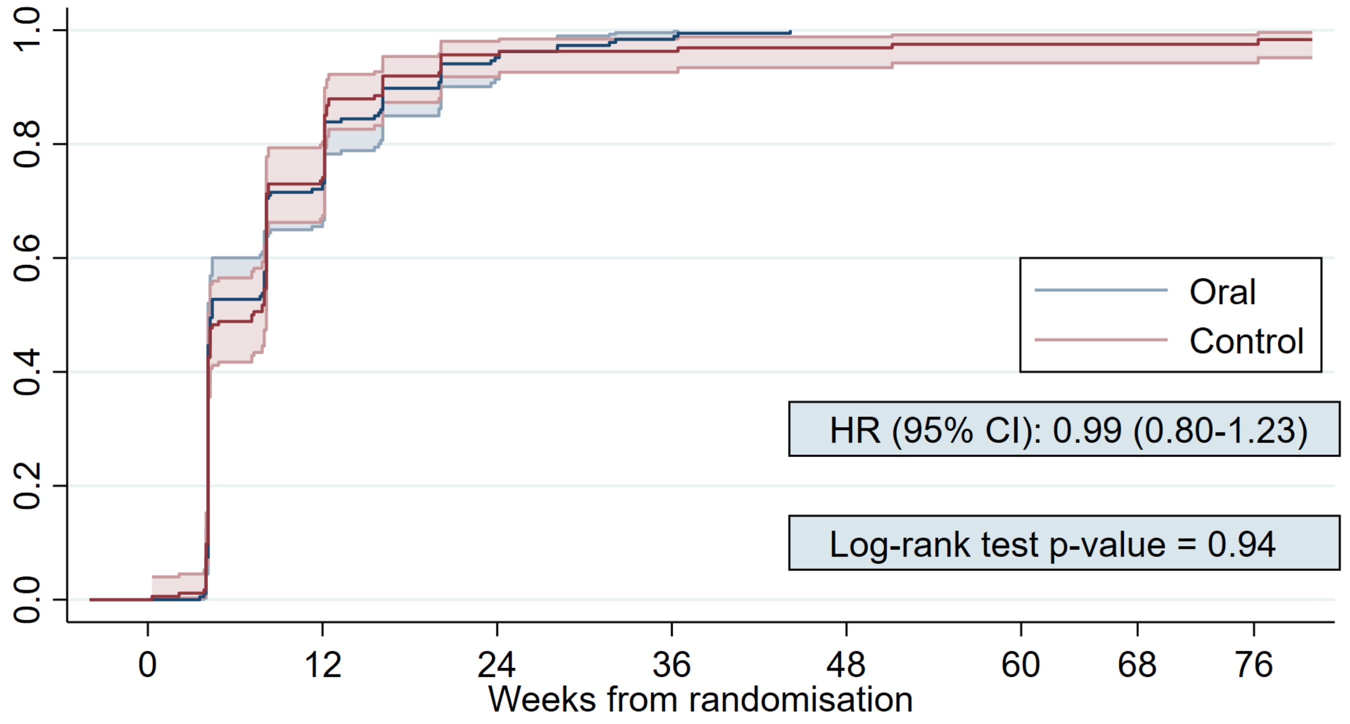

**Oral**								
At-risk	194	52	9	3	0	0	0	0
Censored	0	7	7	7	7	7	7	7
Event	0	135	178	184	187	187	187	187
**Control**								
At-risk	178	46	7	6	5	3	3	3
Censored	0	4	5	5	5	6	6	6
Event	0	128	166	167	168	169	169	169

Table S13: Time to smear conversion: median survival time – Control vs Six-month

	Median survival time (IQR), weeks
**Control**	7.14 (4.14, 12.14)
**Six-month**	8 (4.14, 12.14)

Figure S3: Time to smear conversion: Kaplan-Meier survival estimates and hazard ratio – Control vs Six-month

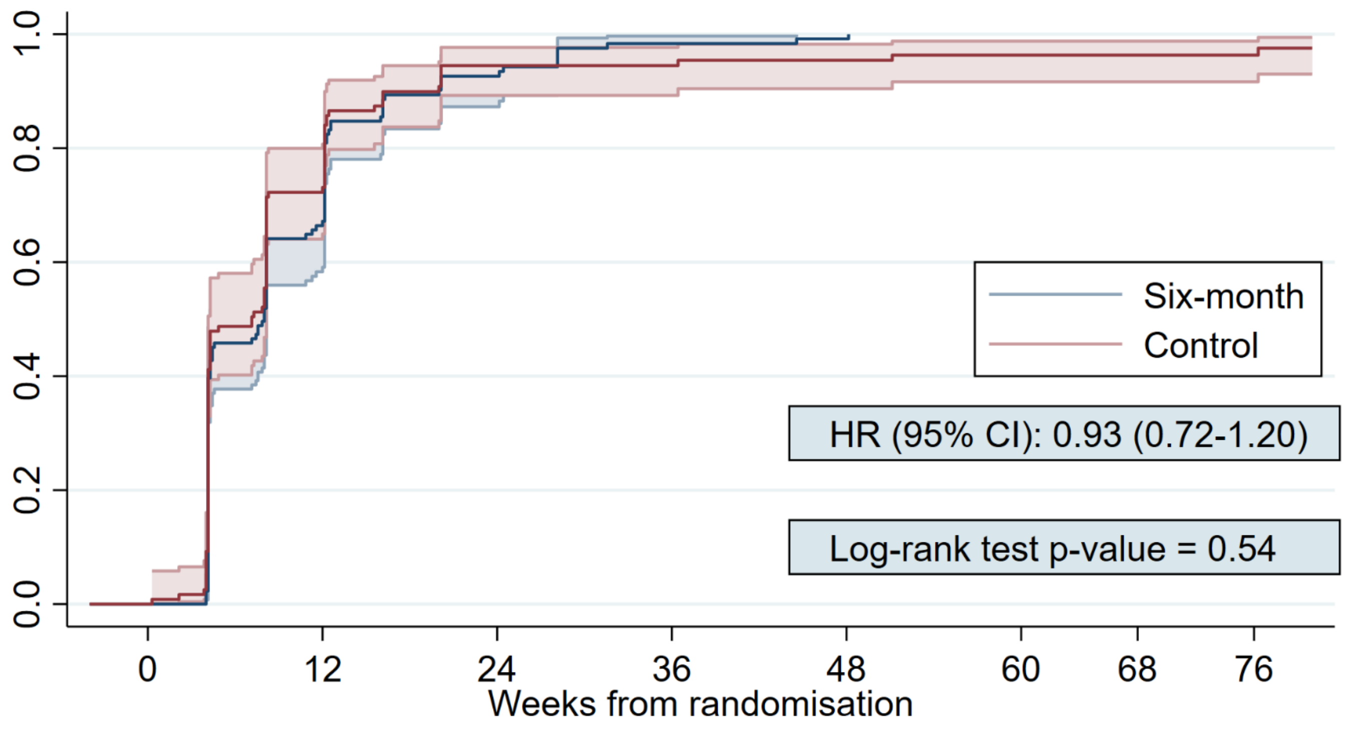

**Six-month**								
At-risk	131	44	9	2	1	0	0	0
Censored	0	0	1	1	1	1	1	1
Event	0	87	121	128	129	130	130	130
**Control**								
At-risk	123	33	6	6	5	3	3	3
Censored	0	4	5	5	5	6	6	6
Event	0	86	112	112	113	114	114	114

Time to sputum culture conversion was defined as the time from randomisation to the first of two consecutive negative culture results, collected on separate days. Patients that never achieved culture conversion were censored at the date of collection of sputum that yielded their last culture result. All patients in the mITT analysis population were included in this analysis.

Table S14: Time to culture conversion: median surival time – Control vs Oral

	Median survival time(IQR), weeks
**Control**	4.14 (4.14, 7.86)
**Oral**	4.14 (4.14, 4.57)

Figure S4: Time to culture conversion: Kaplan-Meier survival estimates and Hazard ratio – Control vs Oral

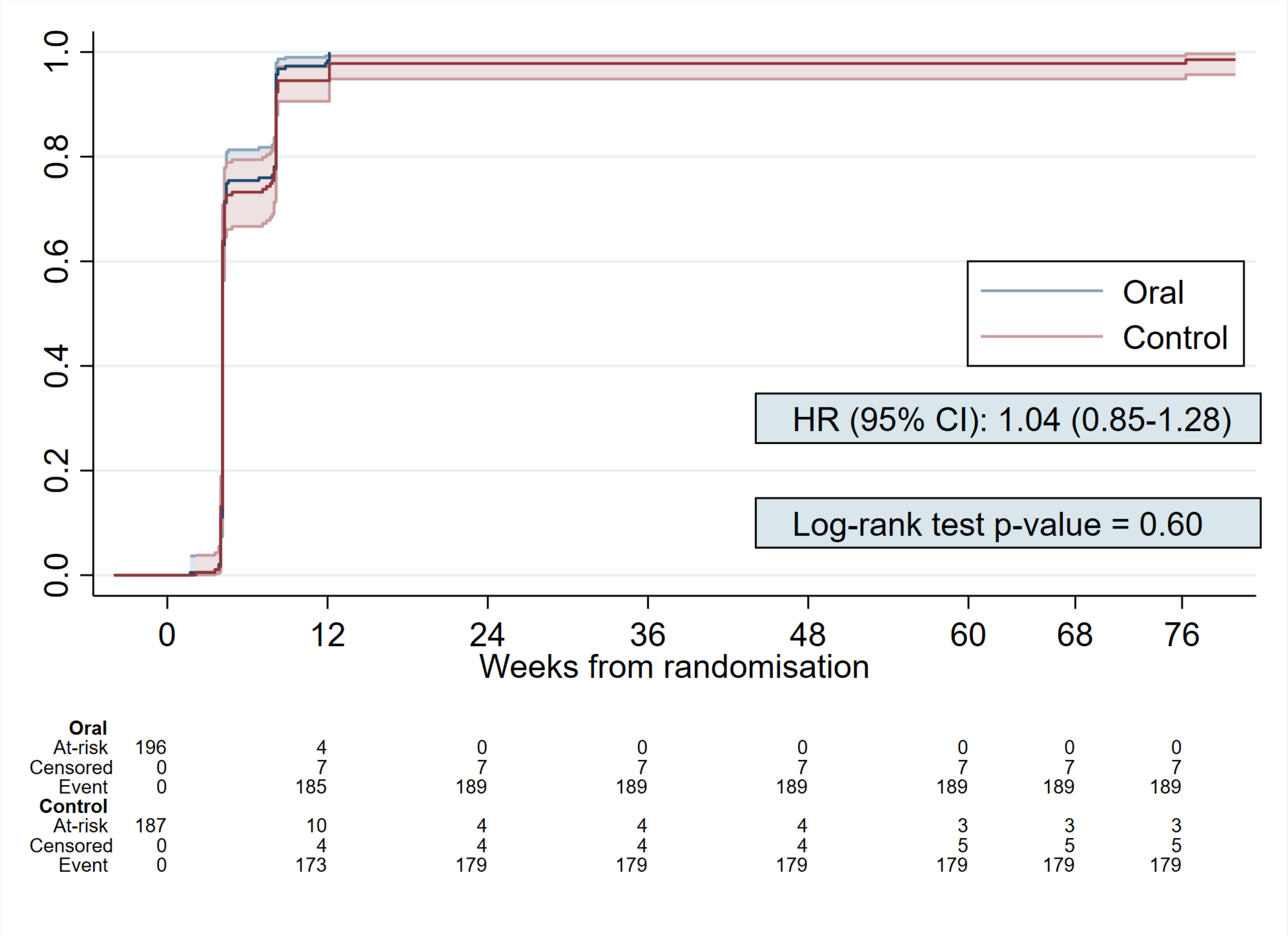

Table S15: Time to culture conversion: median survival time – Control vs. Six-month

	Median survival time (IQR), weeks
**Control**	4.14 (4.14, 4.29)
**Six-month**	4.14 (4.14, 4.29)

Figure S5: Time to culture conversion: Kaplan-Meier survival estimates and Hazard ratio – Control vs. Six-month

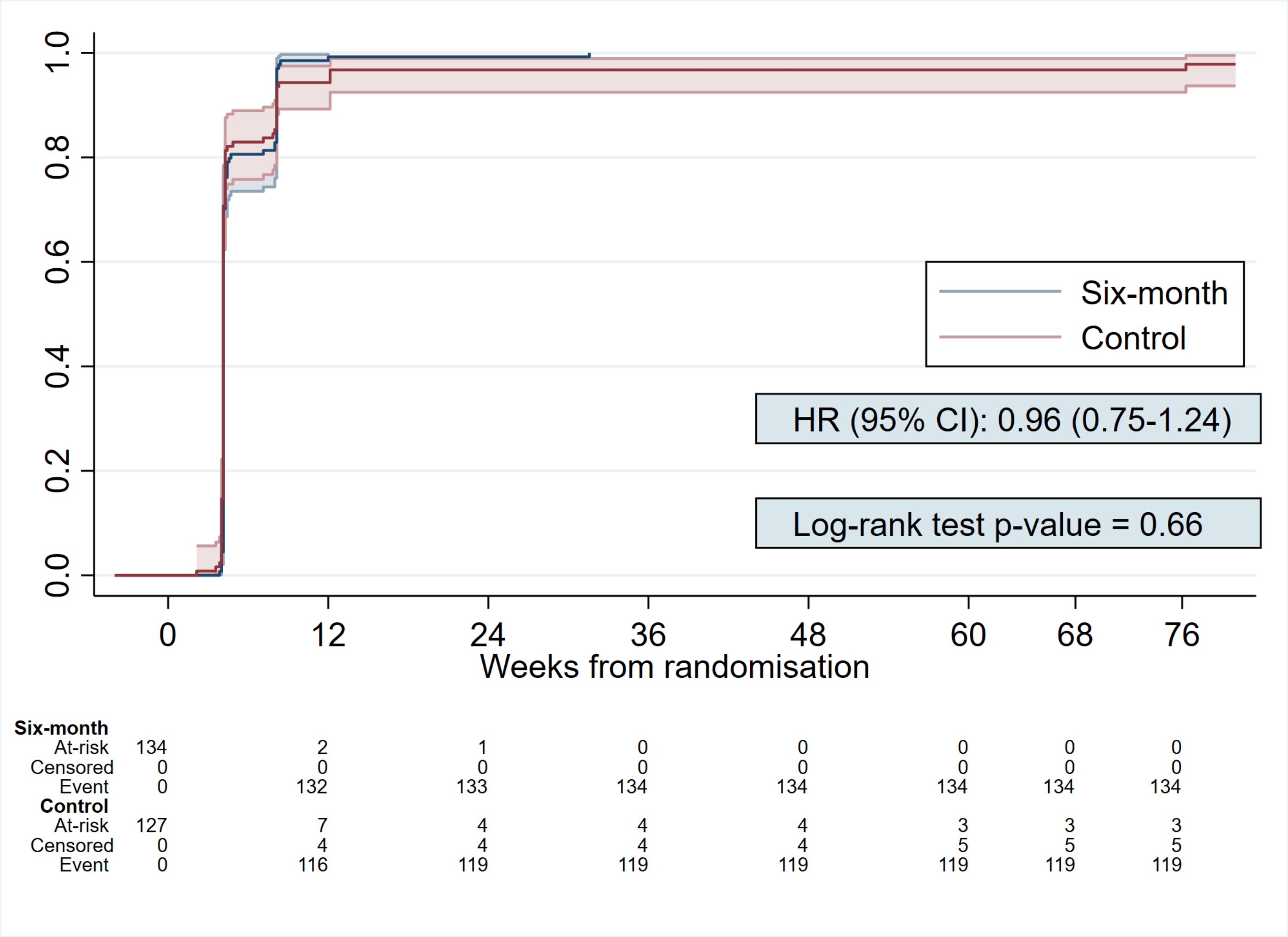

Figure S6 Flat, Sceptical and Expected priors for secondary Bayesian analysis.

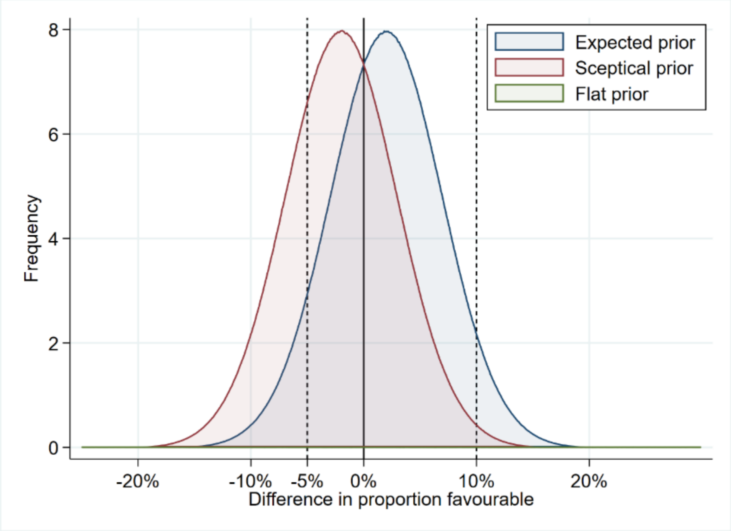

A secondary Bayesian analysis of non-inferiority provides an estimate of the probability that Regimen B has efficacy not much worse than Regimen C for different thresholds of what might be considered 'not much worse'. Following methods described previously,[23] we used Bayesian binomial regression to estimate the distribution of the (unadjusted) difference in the proportion of favourable outcomes between regimens B and C. Gaussian Normal priors will be placed on the intercept term (mean = 0.0 and variance = 100) and on the difference in proportion between regimens (Flat: mean = 0.0 and variance = 100, Sceptical: mean = -0.02 and variance 0.05, and Expected: mean = 0.02 and variance 0.05). The Flat prior is an uninformative prior with very large variance centred around zero representing weak prior information, the Sceptical prior is centred around an absolute 2% increase in proportion of favourable in Regimen B with a smaller variance, and the Expected prior represents the assumptions used in the sample size calculations representing an absolute 2% increase in the proportion of favourable outcomes in regimen C. Initial values for the Markov Chain Monte Carlo algorithm came from estimates from the frequentist binomial regression model. This analysis was carried out on the mITT population.

Figure S7 Results of Bayesian analysis of non-inferiority

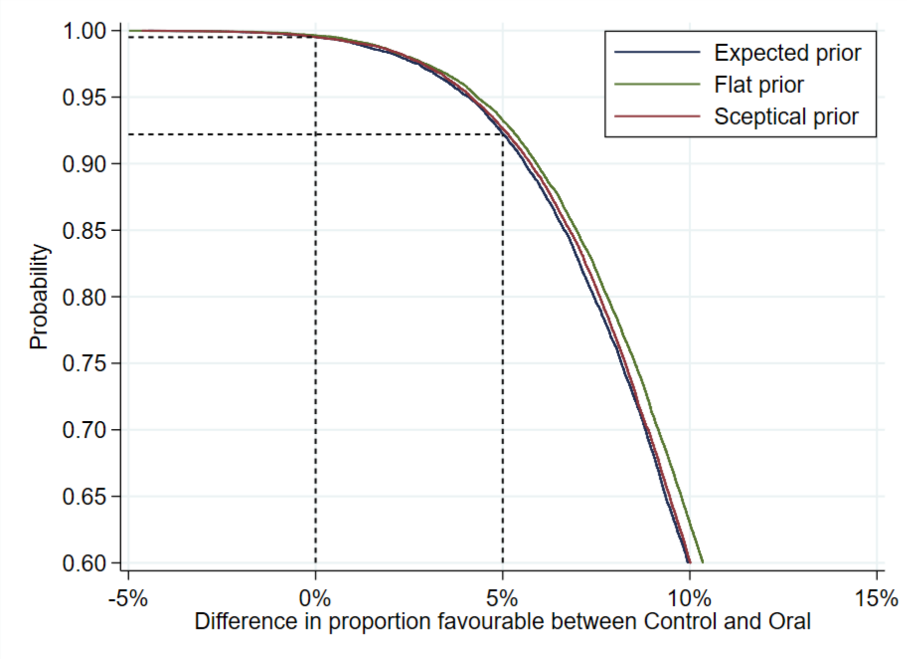

Table S16: Probable cause of death: results of Independent Death Review

Cause of Death from Death Review	Cause of Death from Site Clinician	Control	Oral	Control	Six-month
Cardiac - Arrhythmic: Possible sudden cardiac death	Pulmonary tuberculosis	0	1	0	0
	Sudden death	1	0	1	0
Cardiac - Structural (e.g. IHD, cardiomyopathy, valvular heart disease)	Acute coronary syndrome	0	1	0	0
HIV-related	Respiratory distress	1	0	1	0
Tuberculosis-related	Pulmonary tuberculosis	1	2	0	1
	Respiratory failure	0	1	0	0
Other					
Hypoglycaemia	Neuroglycopenia	0	1	0	0
Hypothermia secondary to alcohol intoxication	Hypothermia	0	0	0	1
Liver cancer	Hepatic cancer	1	0	0	0
Lobar pneumonia	Pneumonia	1	0	0	0
Metastatic carcinoma (probably lung primary)	Lung cancer metastatic	0	1	0	0
Total		5	7	2	2

Six-month participants are compared to the subset of Control participants who were randomised concurrently.

Table S17: Grade 3-4 AE up to week 76: difference in proportion of participants with at least one event between treatment arms

Comparison	Control events/total	Oral/Six-month events/total	Difference (95% CI)	P-value
Control vs Oral	108 (53.5%) / 202	106 (50.2%) / 211	3.3% (-6.3%, 12.9%)	0.50
Control vs Six-month	75 (53.6%) / 140	79 (55.2%) / 143	-1.7% (-13.2%, 9.9%)	0.78

Six-month participants are compared to the subset of Control participants who were randomised concurrently.

Table S18: Grade 3-4 AE on allocated treatment: difference in proportion of participants with at least one event between treatment arms

Comparison	Control events/total	Oral/Six-month events/total	Difference (95% CI)	P-value
Control vs Oral	82 (40.6%) / 202	95 (45.0%) / 211	-4.3% (-13.7%, 5.1%)	0.37
Control vs Six-month	56 (40.0%) / 140	60 (42.0%) / 143	-1.8% (-13.2%, 9.5%)	0.75

Six-month participants are compared to the subset of Control participants who were randomised concurrently.

Table S19: Summary of SAEs by System Organ Class and Preferred Term, up to week 76

System organ class	Preferred term	Control	Oral	Control	Six-month
Any SOC/SMQ	_Any PT	36 (18%)	38 (18%)	27 (19%)	27 (19%)
Blood and lymphatic system disorders	Anaemia	1 (<1%)	0	1 (1%)	0
	_Any PT	1 (<1%)	0	1 (1%)	0
Cardiac disorders	Acute coronary syndrome	0	1 (<1%)	0	0
	_Any PT	0	1 (<1%)	0	0
Gastrointestinal disorders	Abdominal pain	0	0	0	1 (1%)
	Pancreatic pseudocyst	0	0	0	0
	Pancreatitis chronic	0	0	0	0
	Vomiting	0	5 (2%)	0	0
	_Any PT	0	5 (2%)	0	1 (1%)
General disorders and administration site conditions	Asthenia	1 (<1%)	0	1 (1%)	1 (1%)
	Chest pain	0	2 (1%)	0	0
	Hypothermia	0	0	0	1 (1%)
	Pyrexia	0	0	0	0
	Treatment failure	4 (2%)	0	3 (2%)	1 (1%)
	_Any PT	5 (2%)	2 (1%)	4 (3%)	3 (2%)
Hearing and vestibular disorders (SMQ)	Deafness	3 (1%)	3 (1%)	2 (1%)	3 (2%)
	Deafness bilateral	1 (<1%)	0	1 (1%)	1 (1%)
	Deafness unilateral	3 (1%)	3 (1%)	2 (1%)	2 (1%)
	Hypoacusis	0	0	0	1 (1%)
	_Any PT	7 (3%)	6 (3%)	5 (4%)	7 (5%)
Hepatic disorders (SMQ)	Alanine aminotransferase increased	0	1 (<1%)	0	0
	Aspartate aminotransferase increased	1 (<1%)	0	1 (1%)	0
	Drug-induced liver injury	0	1 (<1%)	0	0

System organ class	Preferred term	Control	Oral	Control	Six-month
	Hepatic cancer	1 (<1%)	0	0	0
	Hepatic enzyme increased	0	0	0	0
	Hepatic neoplasm	1 (<1%)	0	0	0
	Hepatitis D	0	1 (<1%)	0	0
	Hepatitis toxic	2 (1%)	1 (<1%)	1 (1%)	1 (1%)
	Transaminases increased	2 (1%)	0	2 (1%)	0
	_Any PT	6 (3%)	4 (2%)	4 (3%)	1 (1%)
Infections and infestations	Anal abscess	1 (<1%)	0	1 (1%)	0
	Bartholinitis	0	1 (<1%)	0	0
	COVID-19	0	0	0	1 (1%)
	Empyema	0	2 (1%)	0	0
	Herpes zoster	1 (<1%)	0	1 (1%)	0
	Pneumonia	2 (1%)	0	1 (1%)	1 (1%)
	Pulmonary tuberculosis	3 (1%)	3 (1%)	2 (1%)	1 (1%)
	Staphylococcal infection	1 (<1%)	0	0	0
	Urinary tract infection	0	1 (<1%)	0	1 (1%)
	_Any PT	8 (4%)	7 (3%)	5 (4%)	3 (2%)
Injury, poisoning and procedural complications	Foot fracture	0	0	0	1 (1%)
	Radius fracture	0	0	0	1 (1%)
	Road traffic accident	1 (<1%)	0	1 (1%)	0
	_Any PT	1 (<1%)	0	1 (1%)	2 (1%)
Investigations	Amylase increased	0	0	0	1 (1%)
	Blood creatinine increased	0	0	0	1 (1%)
	Blood glucose increased	0	1 (<1%)	0	0
	Blood uric acid increased	1 (<1%)	1 (<1%)	0	1 (1%)
	Oxygen saturation decreased	1 (<1%)	0	1 (1%)	0
	SARS-CoV-2 test positive	1 (<1%)	0	1 (1%)	1 (1%)
	_Any PT	3 (1%)	2 (1%)	2 (1%)	4 (3%)
Metabolism and nutrition disorders	Dehydration	0	1 (<1%)	0	0
	Diabetic ketoacidosis	0	0	0	1 (1%)

System organ class	Preferred term	Control	Oral	Control	Six-month
	Hypoglycaemia	0	0	0	1 (1%)
	_Any PT	0	1 (<1%)	0	2 (1%)
Musculoskeletal and connective tissue disorders	Muscle spasms	0	0	0	0
	_Any PT	0	0	0	0
Neoplasms benign, malignant and unspecified (incl cysts and polyps)	Breast cancer	0	1 (<1%)	0	0
	Kaposi's sarcoma	0	1 (<1%)	0	0
	Lung cancer metastatic	0	1 (<1%)	0	1 (1%)
	Prostate cancer	1 (<1%)	0	0	0
	Thyroid cancer	0	1 (<1%)	0	0
	_Any PT	1 (<1%)	4 (2%)	0	1 (1%)
Nervous system disorders	Neuroglycopenia	0	1 (<1%)	0	0
	Polyneuropathy	1 (<1%)	0	0	0
	Seizure	0	0	0	1 (1%)
	Tonic convulsion	0	0	0	1 (1%)
	_Any PT	1 (<1%)	1 (<1%)	0	2 (1%)
Pregnancy, puerperium and perinatal conditions	Abortion	1 (<1%)	0	1 (1%)	0
	Abortion missed	0	1 (<1%)	0	0
	Abortion spontaneous	0	0	0	1 (1%)
	Pregnancy	0	1 (<1%)	0	0
	_Any PT	1 (<1%)	2 (1%)	1 (1%)	1 (1%)
Psychiatric disorders	Acute psychosis	0	0	0	0
	Depression	0	0	0	0
	Major depression	1 (<1%)	0	0	0
	_Any PT	1 (<1%)	0	0	0
Renal and urinary disorders	Urinary retention	1 (<1%)	0	0	0
	_Any PT	1 (<1%)	0	0	0
Respiratory, thoracic and mediastinal disorders	Atelectasis	0	0	0	1 (1%)
	Chronic obstructive pulmonary disease	1 (<1%)	0	1 (1%)	0
	Dyspnoea	3 (1%)	2 (1%)	3 (2%)	0

System organ class	Preferred term	Control	Oral	Control	Six-month
	Pleural effusion	0	1 (<1%)	0	0
	Pneumothorax	0	1 (<1%)	0	1 (1%)
	Respiratory distress	1 (<1%)	0	1 (1%)	0
	Respiratory failure	0	1 (<1%)	0	0
	_Any PT	5 (2%)	4 (2%)	5 (4%)	2 (1%)
Skin and subcutaneous tissue disorders	Stevens-Johnson Syndrome	1 (<1%)	0	1 (1%)	0
	_Any PT	1 (<1%)	0	1 (1%)	0
Social circumstances	Social stay hospitalisation	0	1 (<1%)	0	1 (1%)
	_Any PT	0	1 (<1%)	0	1 (1%)
Surgical and medical procedures	Abortion induced	0	1 (<1%)	0	0
	Appendicectomy	0	1 (<1%)	0	0
	Cholecystectomy	0	0	0	1 (1%)
	Fistulotomy	0	1 (<1%)	0	0
	Genitourinary operation	0	1 (<1%)	0	0
	Hospitalisation	0	2 (1%)	0	0
	_Any PT	0	5 (2%)	0	1 (1%)
Torsade de pointes/QT prolongation (SMQ)	Sudden death	1 (<1%)	0	1 (1%)	0
	Syncope	0	0	0	1 (1%)
	_Any PT	1 (<1%)	0	1 (1%)	1 (1%)
Vascular disorders	Deep vein thrombosis	0	0	0	0
	Embolism venous	0	0	0	0
	Hypotension	0	0	0	1 (1%)
	_Any PT	0	0	0	1 (1%)
_Total	_Total	202	211	140	143

Table S20: Summary of SAEs by System Organ Class and Preferred Term, time on allocated treatment only

System organ class	Preferred term	Control	Oral	Control	Six-month
Any SOC/SMQ	_Any PT	20 (10%)	26 (12%)	14 (10%)	15 (10%)
Blood and lymphatic system disorders	Anaemia	1 (<1%)	0	1 (1%)	0
	_Any PT	1 (<1%)	0	1 (1%)	0
Gastrointestinal disorders	Pancreatic pseudocyst	0	0	0	0
	Pancreatitis chronic	0	0	0	0
	Vomiting	0	3 (1%)	0	0
	_Any PT	0	3 (1%)	0	0
General disorders and administration site conditions	Pyrexia	0	0	0	0
	_Any PT	0	0	0	0
Hearing and vestibular disorders (SMQ)	Deafness	2 (1%)	3 (1%)	1 (1%)	3 (2%)
	Deafness bilateral	1 (<1%)	0	1 (1%)	1 (1%)
	Deafness unilateral	3 (1%)	3 (1%)	2 (1%)	2 (1%)
	Hypoacusis	0	0	0	1 (1%)
	_Any PT	6 (3%)	6 (3%)	4 (3%)	7 (5%)
Hepatic disorders (SMQ)	Alanine aminotransferase increased	0	1 (<1%)	0	0
	Aspartate aminotransferase increased	0	0	0	0
	Drug-induced liver injury	0	1 (<1%)	0	0
	Hepatic enzyme increased	0	0	0	0
	Hepatitis toxic	2 (1%)	1 (<1%)	1 (1%)	0
	Transaminases increased	2 (1%)	0	2 (1%)	0
	_Any PT	4 (2%)	3 (1%)	3 (2%)	0
Infections and infestations	Anal abscess	1 (<1%)	0	1 (1%)	0
	Bartholinitis	0	1 (<1%)	0	0
	Empyema	0	2 (1%)	0	0
	Herpes zoster	1 (<1%)	0	1 (1%)	0
	Pneumonia	0	0	0	1 (1%)

	Staphylococcal infection	1 (<1%)	0	0	0
	Urinary tract infection	0	1 (<1%)	0	0
	_Any PT	3 (1%)	4 (2%)	2 (1%)	1 (1%)
Investigations	Amylase increased	0	0	0	1 (1%)
	Blood creatinine increased	0	0	0	1 (1%)
	Blood glucose increased	0	1 (<1%)	0	0
	Blood uric acid increased	1 (<1%)	1 (<1%)	0	1 (1%)
	_Any PT	1 (<1%)	2 (1%)	0	3 (2%)
Metabolism and nutrition disorders	Dehydration	0	1 (<1%)	0	0
	Diabetic ketoacidosis	0	0	0	1 (1%)
	Hypoglycaemia	0	0	0	1 (1%)
	_Any PT	0	1 (<1%)	0	2 (1%)
Musculoskeletal and connective tissue disorders	Muscle spasms	0	0	0	0
	_Any PT	0	0	0	0
Neoplasms benign, malignant and unspecified (incl cysts and polyps)	Breast cancer	0	1 (<1%)	0	0
	Kaposi's sarcoma	0	1 (<1%)	0	0
	Prostate cancer	1 (<1%)	0	0	0
	_Any PT	1 (<1%)	2 (1%)	0	0
Nervous system disorders	Tonic convulsion	0	0	0	1 (1%)
	_Any PT	0	0	0	1 (1%)
Pregnancy, puerperium and perinatal conditions	Abortion	1 (<1%)	0	1 (1%)	0
	Pregnancy	0	1 (<1%)	0	0
	_Any PT	1 (<1%)	1 (<1%)	1 (1%)	0
Psychiatric disorders	Acute psychosis	0	0	0	0
	Depression	0	0	0	0
	_Any PT	0	0	0	0
Renal and urinary disorders	Urinary retention	1 (<1%)	0	0	0
	_Any PT	1 (<1%)	0	0	0
Respiratory, thoracic and mediastinal disorders	Atelectasis	0	0	0	1 (1%)

	Chronic obstructive pulmonary disease	1 (<1%)	0	1 (1%)	0
	Dyspnoea	1 (<1%)	2 (1%)	1 (1%)	0
	Pleural effusion	0	1 (<1%)	0	0
	Pneumothorax	0	1 (<1%)	0	0
	_Any PT	2 (1%)	3 (1%)	2 (1%)	1 (1%)
Social circumstances	Social stay hospitalisation	0	1 (<1%)	0	0
	_Any PT	0	1 (<1%)	0	0
Surgical and medical procedures	Abortion induced	0	1 (<1%)	0	0
	Appendicectomy	0	1 (<1%)	0	0
	Genitourinary operation	0	1 (<1%)	0	0
	_Any PT	0	3 (1%)	0	0
Torsade de pointes/QT prolongation (SMQ)	Sudden death	1 (<1%)	0	1 (1%)	0
	Syncope	0	0	0	1 (1%)
	_Any PT	1 (<1%)	0	1 (1%)	1 (1%)
Vascular disorders	Deep vein thrombosis	0	0	0	0
	Embolism venous	0	0	0	0
	_Any PT	0	0	0	0
_Total	_Total	202	211	140	143

Table S21: Summary of Grade 3-4 AEs by System Organ Class and Preferred Term, up to 76 weeks

System organ class	Preferred term	Control	Oral	Control	Six-month
Any SOC/SMQ	_Any PT	108 (53%)	106 (50%)	75 (54%)	79 (55%)
Blood and lymphatic system disorders	Anaemia	3 (1%)	1 (<1%)	1 (1%)	4 (3%)
	Neutropenia	0	0	0	1 (1%)
	_Any PT	3 (1%)	1 (<1%)	1 (1%)	5 (3%)
Cardiac disorders	Arrhythmia	0	0	0	1 (1%)
	Cardiac failure congestive	0	1 (<1%)	0	0
	_Any PT	0	1 (<1%)	0	1 (1%)
Eye disorders	Visual impairment	0	1 (<1%)	0	0
	_Any PT	0	1 (<1%)	0	0
Gastrointestinal disorders	Abdominal pain	0	1 (<1%)	0	1 (1%)
	Diarrhoea	0	0	0	1 (1%)
	Nausea	0	0	0	0
	Pancreatitis chronic	0	0	0	0
	Vomiting	1 (<1%)	4 (2%)	1 (1%)	0
	_Any PT	1 (<1%)	4 (2%)	1 (1%)	2 (1%)
General disorders and administration site conditions	Chest pain	0	1 (<1%)	0	1 (1%)
	Injection site reaction	1 (<1%)	0	0	0
	Pyrexia	1 (<1%)	3 (1%)	1 (1%)	1 (1%)
	Treatment failure	2 (1%)	0	1 (1%)	1 (1%)
	_Any PT	4 (2%)	4 (2%)	2 (1%)	3 (2%)
Hearing and vestibular disorders (SMQ)	Deafness	7 (3%)	3 (1%)	6 (4%)	3 (2%)
	Deafness bilateral	2 (1%)	0	1 (1%)	1 (1%)
	Deafness neurosensory	1 (<1%)	0	1 (1%)	0
	Deafness unilateral	10 (5%)	3 (1%)	4 (3%)	2 (1%)
	Dizziness	0	0	0	1 (1%)
	_Any PT	20 (10%)	6 (3%)	12 (9%)	6 (4%)

System organ class	Preferred term	Control	Oral	Control	Six-month
Hepatic disorders (SMQ)	Alanine aminotransferase increased	11 (5%)	8 (4%)	6 (4%)	4 (3%)
	Aspartate aminotransferase increased	19 (9%)	18 (9%)	9 (6%)	3 (2%)
	Bilirubin conjugated increased	0	1 (<1%)	0	0
	Blood bilirubin increased	0	1 (<1%)	0	2 (1%)
	Drug-induced liver injury	0	1 (<1%)	0	0
	Hepatic enzyme increased	2 (1%)	7 (3%)	0	2 (1%)
	Hepatic neoplasm	1 (<1%)	0	0	0
	Hepatitis	0	0	0	1 (1%)
	Hepatitis D	0	1 (<1%)	0	0
	Hepatitis toxic	3 (1%)	2 (1%)	2 (1%)	1 (1%)
	Hepatotoxicity	0	2 (1%)	0	1 (1%)
	Hyperbilirubinaemia	0	1 (<1%)	0	0
	Hypoalbuminaemia	1 (<1%)	0	1 (1%)	0
	Transaminases increased	2 (1%)	0	2 (1%)	2 (1%)
	_Any PT	29 (14%)	32 (15%)	15 (11%)	13 (9%)
Hepatobiliary disorders	Cholelithiasis	0	0	0	1 (1%)
	_Any PT	0	0	0	1 (1%)
Infections and Infestations	Bartholinitis	0	1 (<1%)	0	0
	_Any PT	0	1 (<1%)	0	0
	Anal abscess	1 (<1%)	0	1 (1%)	0
	COVID-19	0	0	0	1 (1%)
	Empyema	0	1 (<1%)	0	0
	Herpes zoster	1 (<1%)	0	1 (1%)	0
	Pneumonia	1 (<1%)	0	1 (1%)	2 (1%)
	Pulmonary tuberculosis	2 (1%)	0	2 (1%)	0
	Staphylococcal infection	1 (<1%)	0	0	0
	_Any PT	6 (3%)	1 (<1%)	5 (4%)	3 (2%)
Injury, poisoning and procedural complications	Foot fracture	0	0	0	1 (1%)

System organ class	Preferred term	Control	Oral	Control	Six-month
	Overdose	0	0	0	2 (1%)
	Radius fracture	0	0	0	1 (1%)
	Road traffic accident	1 (<1%)	0	1 (1%)	0
	_Any PT	1 (<1%)	0	1 (1%)	4 (3%)
Investigations	Amylase increased	4 (2%)	1 (<1%)	4 (3%)	3 (2%)
	Blood bicarbonate decreased	0	0	0	1 (1%)
	Blood bicarbonate increased	0	1 (<1%)	0	0
	Blood cholesterol increased	1 (<1%)	2 (1%)	1 (1%)	0
	Blood creatine increased	0	1 (<1%)	0	0
	Blood creatine phosphokinase MB increased	0	0	0	1 (1%)
	Blood creatine phosphokinase increased	6 (3%)	2 (1%)	6 (4%)	1 (1%)
	Blood creatinine increased	3 (1%)	0	3 (2%)	1 (1%)
	Blood glucose increased	0	1 (<1%)	0	0
	Blood magnesium decreased	3 (1%)	0	2 (1%)	0
	Blood pressure diastolic increased	0	0	0	0
	Blood sodium increased	0	1 (<1%)	0	0
	Blood triglycerides increased	0	1 (<1%)	0	0
	Blood uric acid increased	9 (4%)	9 (4%)	8 (6%)	4 (3%)
	Creatinine renal clearance decreased	1 (<1%)	0	1 (1%)	2 (1%)
	Lipase increased	3 (1%)	1 (<1%)	2 (1%)	4 (3%)
	Oxygen saturation decreased	1 (<1%)	0	1 (1%)	0
	Platelet count decreased	0	1 (<1%)	0	0
	SARS-CoV-2 test positive	0	0	0	1 (1%)
	_Any PT	25 (12%)	17 (8%)	22 (16%)	13 (9%)
Metabolism and nutrition disorders	Abnormal loss of weight	8 (4%)	7 (3%)	3 (2%)	4 (3%)
	Decreased appetite	1 (<1%)	0	1 (1%)	0
	Diabetic ketoacidosis	0	0	0	1 (1%)

System organ class	Preferred term	Control	Oral	Control	Six-month
	Hyperamylasaemia	0	0	0	1 (1%)
	Hyperglycaemia	0	0	0	0
	Hyperkalaemia	0	3 (1%)	0	0
	Hyperlipasaemia	1 (<1%)	0	1 (1%)	0
	Hypernatraemia	1 (<1%)	2 (1%)	1 (1%)	0
	Hyperuricaemia	9 (4%)	15 (7%)	7 (5%)	10 (7%)
	Hypocalcaemia	0	1 (<1%)	0	1 (1%)
	Hypoglycaemia	0	1 (<1%)	0	1 (1%)
	Hypokalaemia	0	0	0	0
	Hypomagnesaemia	0	1 (<1%)	0	0
	Hyponatraemia	2 (1%)	2 (1%)	2 (1%)	1 (1%)
	_Any PT	20 (10%)	27 (13%)	13 (9%)	19 (13%)
Musculoskeletal and connective tissue disorders	Arthralgia	1 (<1%)	0	1 (1%)	1 (1%)
	Back pain	1 (<1%)	0	1 (1%)	0
	Muscle spasms	0	0	0	0
	Myositis	1 (<1%)	0	1 (1%)	0
	_Any PT	2 (1%)	0	2 (1%)	1 (1%)
Neoplasms benign, malignant and unspecified (incl cysts and polyps)	Lung cancer metastatic	0	0	0	1 (1%)
	Oesophageal adenocarcinoma	0	1 (<1%)	0	0
	Prostate cancer	1 (<1%)	0	0	0
	Thyroid cancer	0	1 (<1%)	0	0
	_Any PT	1 (<1%)	2 (1%)	0	1 (1%)
Nervous system disorders	Polyneuropathy	1 (<1%)	0	0	0
	Seizure	0	0	0	1 (1%)
	Tonic convulsion	0	0	0	1 (1%)
	_Any PT	1 (<1%)	0	0	2 (1%)
Pregnancy, puerperium and perinatal conditions	Abortion	1 (<1%)	0	1 (1%)	0
	Abortion missed	0	1 (<1%)	0	0
	_Any PT	1 (<1%)	1 (<1%)	1 (1%)	0

System organ class	Preferred term	Control	Oral	Control	Six-month
Psychiatric disorders	Acute psychosis	0	0	0	0
	Depression	0	0	0	0
	Major depression	1 (<1%)	0	0	0
	_Any PT	1 (<1%)	0	0	0
Renal and urinary disorders	Ketonuria	1 (<1%)	0	1 (1%)	0
	Nephropathy toxic	0	1 (<1%)	0	0
	Urinary retention	1 (<1%)	0	0	0
	_Any PT	2 (1%)	1 (<1%)	1 (1%)	0
Respiratory, thoracic and mediastinal disorders	Atelectasis	0	0	0	1 (1%)
	Chronic obstructive pulmonary disease	1 (<1%)	0	1 (1%)	0
	Cough	1 (<1%)	0	1 (1%)	1 (1%)
	Dyspnoea	4 (2%)	1 (<1%)	4 (3%)	1 (1%)
	Pleural effusion	0	1 (<1%)	0	0
	Pneumothorax	0	0	0	1 (1%)
	_Any PT	5 (2%)	2 (1%)	5 (4%)	3 (2%)
Skin and subcutaneous tissue disorders	Dermatitis allergic	0	1 (<1%)	0	0
	Stevens-Johnson Syndrome	1 (<1%)	0	1 (1%)	0
	_Any PT	1 (<1%)	1 (<1%)	1 (1%)	0
Surgical and medical procedures	Cholecystectomy	0	0	0	1 (1%)
	Fistulotomy	0	1 (<1%)	0	0
	Genitourinary operation	0	1 (<1%)	0	0
	_Any PT	0	2 (1%)	0	1 (1%)
Torsade de pointes/QT prolongation (SMQ)	Electrocardiogram QT prolonged	47 (23%)	50 (24%)	34 (24%)	37 (26%)
	_Any PT	47 (23%)	50 (24%)	34 (24%)	37 (26%)
Vascular disorders	Deep vein thrombosis	1 (<1%)	0	1 (1%)	0
	Embolism venous	0	0	0	0
	Hypertension	1 (<1%)	0	1 (1%)	0
	Hypotension	0	0	0	1 (1%)

System organ class	Preferred term	Control	Oral	Control	Six-month
	_Any PT	2 (1%)	0	2 (1%)	1 (1%)
_Total	_Total	202	211	140	143

Table S22: Summary of Grade 3-4 AEs by System Organ Class and Preferred Term, time on allocated treatment only

System organ class	Preferred term	Control	Oral	Control	Six-month
Any SOC/SMQ	_Any PT	82 (41%)	95 (45%)	56 (40%)	60 (42%)
Blood and lymphatic system disorders	Anaemia	3 (1%)	1 (<1%)	1 (1%)	3 (2%)
	_Any PT	3 (1%)	1 (<1%)	1 (1%)	3 (2%)
Cardiac disorders	Arrhythmia	0	0	0	1 (1%)
	Cardiac failure congestive	0	1 (<1%)	0	0
	_Any PT	0	1 (<1%)	0	1 (1%)
Eye disorders	Visual impairment	0	1 (<1%)	0	0
	_Any PT	0	1 (<1%)	0	0
Gastrointestinal disorders	Abdominal pain	0	1 (<1%)	0	0
	Nausea	0	0	0	0
	Pancreatitis chronic	0	0	0	0

System organ class	Preferred term	Control	Oral	Control	Six-month
	Vomiting	1 (<1%)	3 (1%)	1 (1%)	0
	_Any PT	1 (<1%)	3 (1%)	1 (1%)	0
General disorders and administration site conditions	Chest pain	0	0	0	1 (1%)
	Injection site reaction	1 (<1%)	0	0	0
	Pyrexia	1 (<1%)	2 (1%)	1 (1%)	1 (1%)
	_Any PT	2 (1%)	2 (1%)	1 (1%)	2 (1%)
Hearing and vestibular disorders (SMQ)	Deafness	6 (3%)	3 (1%)	5 (4%)	2 (1%)
	Deafness bilateral	2 (1%)	0	1 (1%)	1 (1%)
	Deafness neurosensory	1 (<1%)	0	1 (1%)	0
	Deafness unilateral	10 (5%)	2 (1%)	4 (3%)	1 (1%)
	Dizziness	0	0	0	1 (1%)
	_Any PT	19 (9%)	5 (2%)	11 (8%)	5 (3%)
Hepatic disorders (SMQ)	Alanine aminotransferase increased	8 (4%)	7 (3%)	5 (4%)	2 (1%)
	Aspartate aminotransferase increased	13 (6%)	13 (6%)	7 (5%)	0
	Bilirubin conjugated increased	0	1 (<1%)	0	0
	Drug-induced liver injury	0	1 (<1%)	0	0
	Hepatic enzyme increased	1 (<1%)	6 (3%)	0	2 (1%)
	Hepatitis	0	0	0	1 (1%)
	Hepatitis toxic	3 (1%)	2 (1%)	2 (1%)	0
	Hepatotoxicity	0	2 (1%)	0	1 (1%)
	Transaminases increased	2 (1%)	0	2 (1%)	2 (1%)
	_Any PT	20 (10%)	26 (12%)	12 (9%)	7 (5%)
Infections and Infestations	Bartholinitis	0	1 (<1%)	0	0
	_Any PT	0	1 (<1%)	0	0
	Anal abscess	1 (<1%)	0	1 (1%)	0
	Empyema	0	1 (<1%)	0	0
	Herpes zoster	1 (<1%)	0	1 (1%)	0
	Pneumonia	0	0	0	2 (1%)

System organ class	Preferred term	Control	Oral	Control	Six-month
	Staphylococcal infection	1 (<1%)	0	0	0
	_Any PT	3 (1%)	1 (<1%)	2 (1%)	2 (1%)
Injury, poisoning and procedural complications	Overdose	0	0	0	2 (1%)
	_Any PT	0	0	0	2 (1%)
Investigations	Amylase increased	3 (1%)	1 (<1%)	3 (2%)	0
	Blood bicarbonate decreased	0	0	0	1 (1%)
	Blood bicarbonate increased	0	1 (<1%)	0	0
	Blood cholesterol increased	1 (<1%)	1 (<1%)	1 (1%)	0
	Blood creatine phosphokinase increased	2 (1%)	2 (1%)	2 (1%)	0
	Blood creatinine increased	2 (1%)	0	2 (1%)	1 (1%)
	Blood glucose increased	0	1 (<1%)	0	0
	Blood magnesium decreased	2 (1%)	0	1 (1%)	0
	Blood pressure diastolic increased	0	0	0	0
	Blood triglycerides increased	0	1 (<1%)	0	0
	Blood uric acid increased	8 (4%)	9 (4%)	7 (5%)	4 (3%)
	Creatinine renal clearance decreased	1 (<1%)	0	1 (1%)	2 (1%)
	Lipase increased	2 (1%)	0	1 (1%)	0
	_Any PT	16 (8%)	14 (7%)	13 (9%)	6 (4%)
Metabolism and nutrition disorders	Abnormal loss of weight	5 (2%)	6 (3%)	1 (1%)	2 (1%)
	Decreased appetite	1 (<1%)	0	1 (1%)	0
	Diabetic ketoacidosis	0	0	0	1 (1%)
	Hyperamylasaemia	0	0	0	1 (1%)
	Hyperglycaemia	0	0	0	0
	Hyperkalaemia	0	1 (<1%)	0	0
	Hyperlipasaemia	1 (<1%)	0	1 (1%)	0
	Hypernatraemia	1 (<1%)	1 (<1%)	1 (1%)	0
	Hyperuricaemia	9 (4%)	14 (7%)	7 (5%)	10 (7%)

System organ class	Preferred term	Control	Oral	Control	Six-month
	Hypocalcaemia	0	1 (<1%)	0	0
	Hypoglycaemia	0	1 (<1%)	0	1 (1%)
	Hypokalaemia	0	0	0	0
	Hypomagnesaemia	0	1 (<1%)	0	0
	Hyponatraemia	1 (<1%)	2 (1%)	1 (1%)	0
	_Any PT	16 (8%)	23 (11%)	10 (7%)	15 (10%)
Musculoskeletal and connective tissue disorders	Arthralgia	0	0	0	0
	Muscle spasms	0	0	0	0
	Myositis	1 (<1%)	0	1 (1%)	0
	_Any PT	1 (<1%)	0	1 (1%)	0
Neoplasms benign, malignant and unspecified (incl cysts and polyps)	Prostate cancer	1 (<1%)	0	0	0
	_Any PT	1 (<1%)	0	0	0
Nervous system disorders	Tonic convulsion	0	0	0	1 (1%)
	_Any PT	0	0	0	1 (1%)
Pregnancy, puerperium and perinatal conditions	Abortion	1 (<1%)	0	1 (1%)	0
	_Any PT	1 (<1%)	0	1 (1%)	0
Psychiatric disorders	Acute psychosis	0	0	0	0
	Depression	0	0	0	0
	_Any PT	0	0	0	0
Renal and urinary disorders	Ketonuria	1 (<1%)	0	1 (1%)	0
	Nephropathy toxic	0	1 (<1%)	0	0
	Urinary retention	1 (<1%)	0	0	0
	_Any PT	2 (1%)	1 (<1%)	1 (1%)	0
Respiratory, thoracic and mediastinal disorders	Atelectasis	0	0	0	1 (1%)
	Chronic obstructive pulmonary disease	1 (<1%)	0	1 (1%)	0
	Cough	0	0	0	1 (1%)
	Dyspnoea	1 (<1%)	1 (<1%)	1 (1%)	1 (1%)
	Pleural effusion	0	1 (<1%)	0	0

System organ class	Preferred term	Control	Oral	Control	Six-month
	_Any PT	2 (1%)	2 (1%)	2 (1%)	2 (1%)
Skin and subcutaneous tissue disorders	Dermatitis allergic	0	1 (<1%)	0	0
	_Any PT	0	1 (<1%)	0	0
Surgical and medical procedures	Genitourinary operation	0	1 (<1%)	0	0
	_Any PT	0	1 (<1%)	0	0
Torsade de pointes/QT prolongation (SMQ)	Electrocardiogram QT prolonged	31 (15%)	44 (21%)	23 (16%)	26 (18%)
	_Any PT	31 (15%)	44 (21%)	23 (16%)	26 (18%)
Vascular disorders	Deep vein thrombosis	1 (<1%)	0	1 (1%)	0
	Embolism venous	0	0	0	0
	Hypertension	1 (<1%)	0	1 (1%)	0
	_Any PT	2 (1%)	0	2 (1%)	0
_Total	_Total	202	211	140	143

Table S23: Proportions of participants with change in trial regimen following AE

Comparison	Control events/total	Oral/Six-month events/total	Difference (95% CI)	P-value
**Control vs Oral**	61 (30.2%) / 202	37 (17.5%) / 211	12.7% (4.6%, 20.8%)	0.0022
**Control vs Six-month**	41 (29.3%) / 140	26 (18.2%) / 143	11.0% (1.2%, 20.9%)	0.028

Six-month participants are compared to the subset of Control participants who were randomised concurrently.

Table S24: Changes in regimen following AE, by allocated regimen

Drug action	Type of AE	Control	Oral	Control	Six-month
**Total**		202	211	140	143
**Permanent Stop**	Hearing and vestibular disorders	16 (8%)	0	11 (8%)	1 (1%)
	Hepatic disorders	6 (3%)	8 (4%)	3 (2%)	1 (1%)
	QT prolongation	6 (3%)	2 (1%)	5 (4%)	1 (1%)
	Other	9 (4%)	5 (2%)	8 (6%)	5 (3%)
	Total	37 (18%)	15 (7%)	27 (19%)	8 (6%)
**Dose or Freq Change**	Hearing and vestibular disorders	3 (1%)	0	2 (1%)	3 (2%)
	QT prolongation	1 (<1%)	0	1 (1%)	0
	Other	3 (1%)	0	3 (2%)	2 (1%)
	Total	7 (3%)	0	6 (4%)	5 (3%)
**Withholding**	Hearing and vestibular disorders	2 (1%)	0	1 (1%)	1 (1%)
	Hepatic disorders	8 (4%)	14 (7%)	3 (2%)	3 (2%)
	QT prolongation	4 (2%)	3 (1%)	2 (1%)	4 (3%)
	Other	0	5 (2%)	0	3 (2%)
	Total	14 (7%)	22 (10%)	6 (4%)	11 (8%)

Participants may have modified their treatment on more than one occasion. In this table, events were prioritised in the order: permanent stop, dose or frequency change, withholding, and participants appear only once.
Six-month participants are compared to the subset of Control participants who were randomised concurrently.

Figure S8: Plot of change from baseline in mean QT interval (ms) by treatment arm, over time from randomisation

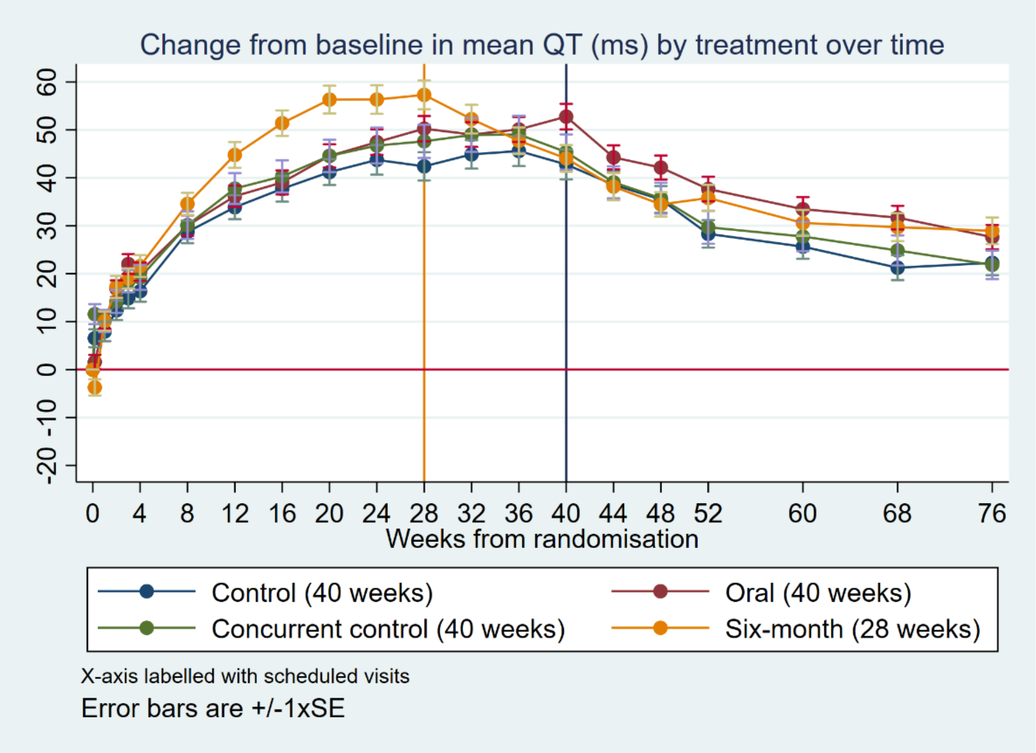

Figure S9: Plot of change from baseline in mean QTcF interval (ms) by treatment arm, over time from randomisation

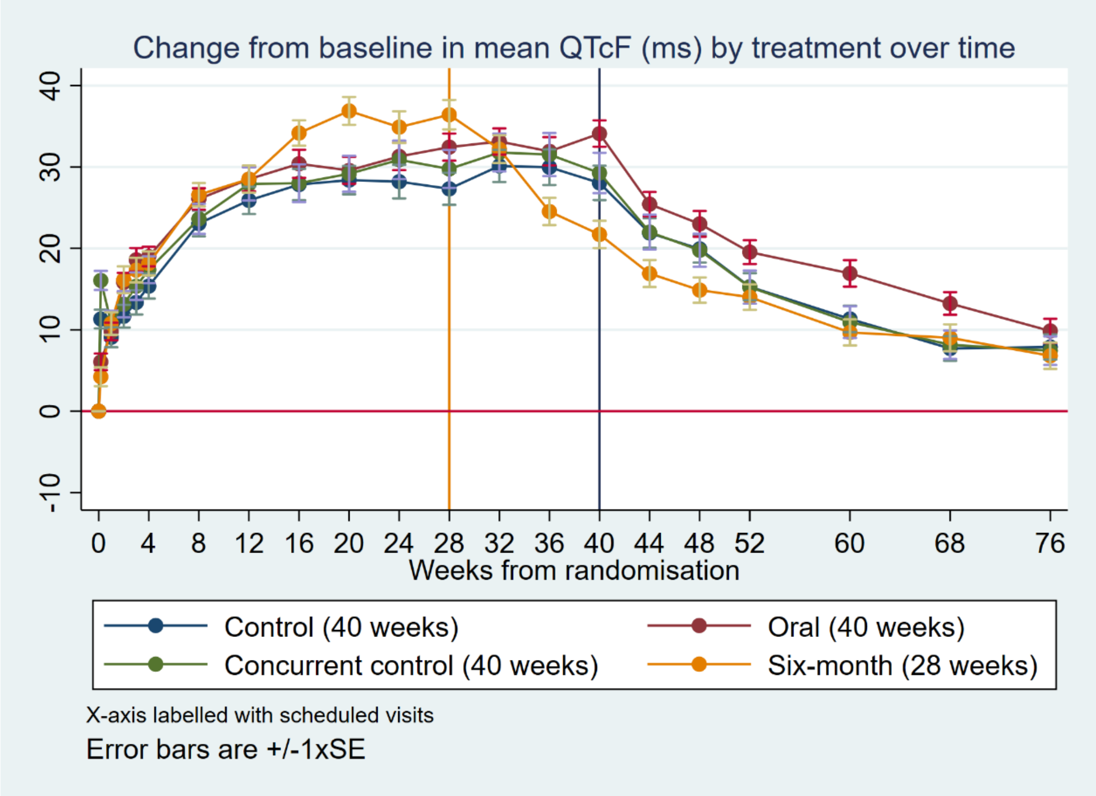

References

1. Chiang CY, Lee JJ, Chien ST, et al. Glycemic control and radiographic manifestations of tuberculosis in diabetic patients. *PLoS One*. 2014;9(4):e93397. doi:10.1371/journal.pone.0093397
2. Steingart KR, Henry M, Ng V, et al. Fluorescence versus conventional sputum smear microscopy for tuberculosis: a systematic review. *The Lancet infectious diseases*. 2006;6(9):570-581.
3. World Health Organisation. WHO Laboratory Services in Tuberculosis Control. Part II: Microscopy. WHO/TB/98.258. 1998;
4. Anthony R, Kolk A, Kuijper S, Klatser P. Light emitting diodes for auramine O fluorescence microscopic screening of Mycobacterium tuberculosis. *Int J Tuberc Lung Dis*. 2006;10(9):1060-1062.
5. Van Deun A, Maug A, Hossain A, Gumusboga M, De Jong B. Fluorescein diacetate vital staining allows earlier diagnosis of rifampicin-resistant tuberculosis. *Int J Tuberc Lung Dis*. 2012;16(9):1174-1179.
6. Salim H, Aung K, Hossain M, Van Deun A. Early and rapid microscopy-based diagnosis of true treatment failure and MDR-TB. *Int J Tuberc Lung Dis*. 2006;10(11):1248-1254.
7. Kudoh S, Kudoh T. A simple technique for culturing tubercle bacilli. *Bull World Health Organ*. 1974;51(1):71.
8. Nathavitharana RR, Hillemann D, Schumacher SG, et al. Multicenter noninferiority evaluation of Hain GenoType MTBDR plus version 2 and Nipro NTM+ MDRTB line probe assays for detection of rifampin and isoniazid resistance. *J Clin Microbiol*. 2016;54(6):1624-1630.
9. Chakravorty S, Simmons AM, Rowneki M, et al. The new Xpert MTB/RIF Ultra: improving detection of Mycobacterium tuberculosis and resistance to rifampin in an assay suitable for point-of-care testing. *MBio*. 2017;8(4):e00812-17.
10. World Health Organisation. *Automated real-time nucleic acid amplification technology for rapid and simultaneous detection of tuberculosis and rifampicin resistance: Xpert MTB*. 2013. 9241506334.
11. Abe C, Hirano K, Tomiyama T. Simple and rapid identification of the Mycobacterium tuberculosis complex by immunochromatographic assay using anti-MPB64 monoclonal antibodies. *J Clin Microbiol*. 1999;37(11):3693-3697.
12. Tagliani E, Cabibbe AM, Miotto P, et al. Diagnostic performance of the new version (v2. 0) of GenoType MTBDR sl assay for detection of resistance to fluoroquinolones and second-line injectable drugs: a multicenter study. *J Clin Microbiol*. 2015;53(9):2961-2969.
13. Nepali S, Ghimire P, Khadka D, Acharya S. Selective inhibition of Mycobacterium tuberculosis by para-nitrobenzoic acid (PNB) used in Lowenstein-Jensen medium. *SAARC Journal of Tuberculosis, Lung Diseases and HIV/AIDS*. 2008;5(1):25-28.
14. Canetti G, Rist N, Grosset J. Measurement of sensitivity of the tuberculous bacillus to antibacillary drugs by the method of proportions. Methodology, resistance criteria, results and interpretation. *Revue de tuberculose et de pneumologie*. 1963;27:217-272.
15. World Health Organisation. Technical manual for drug susceptibility testing of medicines used in the treatment of tuberculosis. 2018;
16. World Health Organisation. Updated interim critical concentrations for first-line and second-line DST. *WHO, Geneva, Switzerland*. 2012;
17. Sanger F, Nicklen S, Coulson AR. DNA sequencing with chain-terminating inhibitors. *Proceedings of the National Academy of Sciences*. 1977;74(12):5463-5467.
18. Supply P, Lesjean S, Savine E, Kremer K, Van Soolingen D, Locht C. Automated high-throughput genotyping for study of global epidemiology of Mycobacterium tuberculosis based on mycobacterial interspersed repetitive units. *J Clin Microbiol*. 2001;39(10):3563-3571.
19. Supply P, Allix C, Lesjean S, et al. Proposal for standardization of optimized mycobacterial interspersed repetitive unit-variable-number tandem repeat typing of Mycobacterium tuberculosis. *J Clin Microbiol*. 2006;44(12):4498-4510.
20. Martin A, Camacho M, Portaels F, Palomino JC. Resazurin microtiter assay plate testing of Mycobacterium tuberculosis susceptibilities to second-line drugs: rapid, simple, and inexpensive method. *Antimicrob Agents Chemother*. 2003;47(11):3616-3619.
21. World Health Organisation. *WHO consolidated guidelines on drug-resistant tuberculosis treatment. Geneva: World Health Organisation; 2019. Licence: CC BY-NC-SA 3.0 IGO.* 2019.
22. Phillips PPJ, Van Deun A, Ahmed S, et al. Investigation of the efficacy of the short regimen for rifampicin-resistant TB from the STREAM trial. *BMC Medicine*. 2020/11/04 2020;18(1):314. doi:10.1186/s12916-020-01770-z

23. Nunn AJ, Phillips PP, Meredith SK, et al. A trial of a shorter regimen for rifampin-resistant tuberculosis. *N Engl J Med*. 2019;380(13):1201-1213.
